# Unravelling the Genetic Basis of Insect Resistance in Maize: Progress and Future Challenges

**DOI:** 10.3390/plants15193005

**Published:** 2026-09-30

**Authors:** Sunandan Swain, Rohit, Ankushpreet Singh, Sanjay Kumar, Gautam Chhabra, Jawala Jindal, Tosh Garg, Surinder K. Sandhu, Yogesh Vikal, Ha Van Gioi, Rumesh Ranjan

**Affiliations:** 1 Department of Plant Breeding and Genetics, Punjab Agricultural University, Ludhiana 141004, Indiarohitbscagriculture2019@gmail.com (R.);; 2 School of Agricultural Biotechnology, Punjab Agricultural University, Ludhiana 141004, India; 3 Maize Germplasm Resources Department, Maize Research Institute, Hanoi 153300, Vietnam; 4 VinUni Big Data Research Institute, VinUniversity, Hanoi 10000, Vietnam

**Keywords:** insect resistance, host plant resistance, biochemical approach, molecular approach, sustainable crop protection, maize

## Abstract

Maize, the queen of cereals, is generally affected by several insect pests throughout its lifecycle and by post-harvest pests, which affects yield and grain quality, resulting in heavy reliance on chemical insecticides. Although host-plant resistance is environmentally friendly, its polygenic inheritance, genotype-by-environment interactions, the defence–yield trade-off, and pests’ ability to adapt quickly make it imperative for researchers to understand the genetic architecture in order to initiate insect resistance breeding programs in maize. This review critically summarises the genetic, biochemical, and physiological baseis of insect resistance in maize, including mechanisms of antixenosis, antibiosis, tolerance, structural barriers, benzoxazinoids, protease inhibitors, and herbivore-induced volatiles and jasmonate-mediated signalling. It also assesses the advancement of germplasm screening, conventional and marker-assisted breeding, quantitative trait locus mapping, genome-wide association studies, genomic selection, clustered regularly interspaced short palindromic repeats (CRISPR) and CRISPR-associated protein (Cas) genome editing, and transgenic *Bacillus thuringiensis* (Bt) technologies. It seems that for durable resistance to be achieved, it will be necessary to combine complementary defence mechanisms rather than relying on single loci or toxins. Future progress needs to incorporate landrace and wild-relative diversity into high-throughput phenotyping, pangenomics, multi-omics, multi-environment prediction, functional validation, and resistance management. This integrated approach can facilitate the development of higher-yielding, climate-smart, multi-pest-resistant maize and minimize pesticide use and associated environmental impacts.

## 1. Introduction

Maize (*Zea mays* L.) is a versatile, widely cultivated, and economically important crop that serves as a major source of food, feed, and industrial raw materials worldwide [1].

However, the productivity and quality of maize production are limited by several biotic and abiotic stresses, among which the insect pests are the major concern, including many complex combinations of diverse phytophagous insect pests that attack maize from the seedling stage through post-harvest storage. Key examples include the fall armyworm (*Spodoptera frugiperda* J.E. Smith), which has rapidly expanded across Africa and Asia due to its wide host range, high reproductive capacity, and potential for multiple infestations within a single crop cycle [2,3]; stem borers such as *Busseola fusca* (Fuller) and *Chilo partellus* (Swinhoe), which damage leaves, stems, and reproductive tissues, thereby weakening plants and reducing grain set [4,5]; ear-feeding insects, which directly consume kernels and predispose grain to fungal infection and mycotoxin contamination [6]; and sap-feeding pests like the corn leaf aphid (*Rhopalosiphum maidis* Fitch), which deplete assimilates, disrupt reproductive growth, and transmit viruses such as maize dwarf mosaic virus (MDMV) and sugarcane mosaic virus (SCMV) [7,8,9].

Moreover, up to 40% of the losses continue after harvest, especially from storage pests [10]. Maize weevil (*Sitophilus zeamais* (Motschul’skii)) is one of the most destructive storage pests of maize, causing losses due to kernel perforation, weight loss, reduced seed viability, contamination, and deterioration in nutritional and market quality. These impacts are especially high when production systems lack suitable facilities for drying, storage, sanitation, and pest exclusion [11,12,13]. Therefore, the impacts of insect pests on maize production are multifaceted and interdependent, including direct consumption of plant tissues, interference with plant physiology, indirect pest-related impacts such as pathogen transmission and facilitation, contamination of harvested grain, and higher pest-control costs.

Traditionally, synthetic insecticides have been used to manage maize insect pests, and transgenic insecticidal traits have been used, primarily in specific production systems worldwide. While these technologies offer good pest suppression, their frequent and improper use has led to increased production costs, pesticide-related risks to farmers and consumers, impacts on non-target organisms, environmental contamination and selection for pesticide-resistant insect populations [14,15]. These restrictions are particularly important for smallholder production systems where information on pest monitoring, suitable use of equipment, protective clothing and recommended insecticides may be limited.

Integrated pest management (IPM) relies on a combination of several tactics to address insect pests in maize, such as cultural practices, biological control, mating-disruption technologies, synthetic pesticides, agroecological practices, pest monitoring, resistance management and host-plant resistant varieties. The adoption of these technologies, however, is not uniform among farm populations due to regulatory issues and poor access across several countries, especially the sub-Saharan African region [2]. Host-plant resistance (HPR) is the natural ability of a plant to reduce pest colonization, damage, or yield loss through three main mechanisms: antixenosis, antibiosis, and tolerance [16]. The mechanisms can act alone or in combination to establish the final resistance phenotype [16,17].

Maize HPR is a combination of a myriad of morphological, biochemical, physiological, and molecular traits. One example of the structural characteristics that might limit feeding or tissue digestibility is toughness. Biochemical defences repel feeding or disrupt insect development, and can also produce other volatiles when attacked by herbivores, attracting predators and parasitoids and effectively defending the crop indirectly. The interference among these responses is mediated by interactions among various signalling pathways, including jasmonic acid, salicylic acid and ethylene, and their transcriptional regulators [9,14,18,19]. HPR is quantitative in nature and controlled by several genes with a small to moderate effect, which can vary between tissues, developmental stages, pest species and environments.

Conventional screening and selection efforts have identified useful resistance in elite inbreds, landraces, exotic germplasm and teosinte-derived materials. Breeding progress is, however, often hampered due to labour-intensive and variable phenotyping, genotype × environment interactions, linkage drag, limited genetic diversity in elite breeding pools and the requirement for resistance combined with high yield, agronomic adaptation, grain quality and farmer-preferred traits [19,20]. In addition, there may be differences in resistance within insect biotypes or populations, and the efficacy of a single resistance gene or protein may be suppressed by continued evolution of a pest.

The use of advanced molecular breeding strategies, quantitative trait locus (QTL) mapping, genome-wide association studies (GWAS), high-density genotyping, marker-assisted selection (MAS), and genomic prediction enables the identification of regions containing genes for resistance and the more efficient accumulation of desirable alleles [14,21]. Further multi-omic approaches, such as transcriptomic, proteomic, and metabolomic methods, can also identify defence pathways, regulatory networks, and candidate genes that cannot be easily identified by phenotype-based evaluation. Other, mechanistically distinct, ways to control insects notably include the use of transgenic Bt traits and RNA interference (RNAi), techniques which also complement developing resistance varieties [22,23]. Genome-editing technologies such as CRISPR/Cas enable candidate-gene validation, targeted knockout or precise modification of susceptibility genes and endogenous defence pathways, and the introduction of multiple resistance-associated changes [24,25].

However, reliance on a single gene, metabolite or insecticidal protein will not lead to durable resistance. Complementary defence mechanisms, conservation of genetic diversity, multi-environment validation, monitoring of pest adaptation and integration with other IPM and resistance management strategies will be required for effective deployment.

This review compiles recent advances in insect resistance in maize, spanning genetic, morphological, biochemical, physiological, molecular and other perspectives, with particular mention of fall armyworm (FAW) and other key insect pests of maize in Asia and Africa. It briefly introduces the concepts of resistant germplasm, conventional breeding, QTL mapping, marker-assisted and genomic selection, transgenic and RNA-based tools, and the development of gene-editing tools (CRISPR-Cas). It identifies key challenges in breeding resistant cultivars, including polygenetic inheritance, genotype–environment interactions, phenotyping accuracy, the agronomic trade-offs, and pest adaptation. Future priorities include incorporation of landraces and wild relatives, high-throughput phenotyping, pangenomics, multi-omics, predictive breeding, functional validation and IPM to create durable, climate-resilient, multi-pest-resistant maize varieties.

## 2. Mechanisms of Resistance in Maize

Host-plant resistance refers to a property of plant species that prevents, tolerates or recovers from injury inflicted by insect populations, thereby decreasing the successful utilization of these plants for insect food and reproduction.

**Figure 1:**
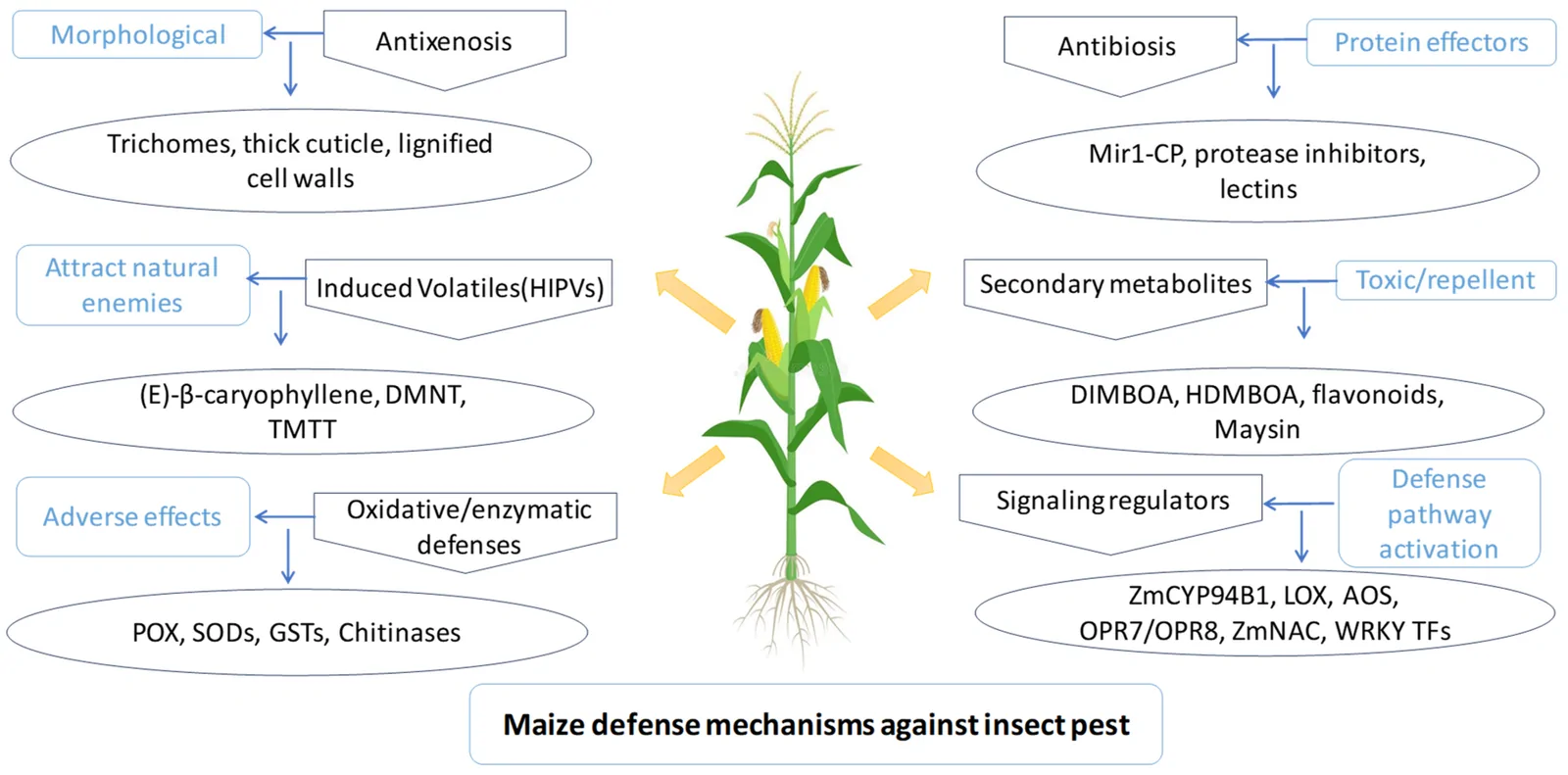
Mechanisms of insect resistance in the host. The figure summarizes antixenosis, antibiosis, tolerance, direct and indirect defences, and the associated molecular regulatory pathways. All abbreviations and gene/protein acronyms used in the figure are defined in the following. Abbreviations: AOS, allene oxide synthase; DIMBOA, 2,4-dihydroxy-7-methoxy-1,4-benzoxazin-3-one; DMNT, (E)-4,8-dimethyl-1,3,7-nonatriene; GSTs, glutathione S-transferases; HDMBOA, 2-hydroxy-4,7-dimethoxy-1,4-benzoxazin-3-one; HIPVs, herbivore-induced plant volatiles; LOX, lipoxygenase; Mir1-CP, maize insect resistance 1 cysteine protease; OPR7/OPR8, 12-oxophytodienoate reductases 7 and 8; POX, peroxidase; SODs, superoxide dismutases; TFs, transcription factors; TMTT, (E,E)-4,8,12-trimethyl-1,3,7,11-tridecatetraene; WRKY, WRKY transcription factor family; ZmCYP94B1, *Zea mays* cytochrome P450 94B1; ZmNAC, *Zea mays* NAC transcription factor.

It comprises a wide range of genetically inherent qualities (biophysical and biochemical) manifested through three possible mechanisms, namely, antixenosis, antibiosis, and tolerance [16]. These mechanisms can occur singly or in combination and can differ between plant tissues, developmental stages, insect species and environments [17,26,27]. Host defences can be either constitutive or inducible, either direct or indirect. Direct defences include physical structures, specialised metabolites, and feeding-deterrent proteins that interfere with insect digestion and nutrient uptake. Indirect defences include herbivore-induced plant volatiles (HIPVs) that attract predators and parasitoids; a pioneering, landmark study of maize showed that herbivore feeding triggers the release of terpenoids that are detected by host-seeking parasitoids [28]. At the signalling level, jasmonates produced via the lipoxygenase-dependent oxylipin pathway play a key role in maize anti-herbivore defence, while ethylene and salicylic acid regulate the intensity and timing of downstream responses [18,29]. Figure 1 represents a summary of the principal direct, indirect, and regulatory systems involved in maize insect defence.

### 2.1. Antixenosis (Non-Preference)

Antixenosis is a pre-ingestive resistance mechanism that involves plant characteristics that decrease attraction, host acceptance, feeding, oviposition and shelter selection by the insect [30,31]. It is affected by physical and chemical properties such as plant surface morphology, tissue toughness, cuticular properties, and the presence or absence of volatile or non-volatile compounds that affect insect host recognition and preference [32]. For example, FAW neonates fail to establish on the resistant line CML71 because resistance is localized in leaf tissues [27]. Some compounds, such as maysin-a C-glycosyl flavone in corn silks, primarily reduce pest feeding and oviposition but can cause post-ingestion toxic effects, creating an overlap with antibiosis [33,34]. Furthermore, high hemicellulose, low protein content, leaf toughness, jasmonic acid, (E)-β-caryophyllene, cuticular lipids, and silica deposits also reduce plant attractiveness/preference to pests [34,35].

### 2.2. Antibiosis

Antibiosis is an effect of host-plant constituents that slows or inhibits pest growth or survival upon ingestion. It causes reductions in insect fertility and fecundity, delayed development, reduced body weight, deformities, abnormal behaviour, or even death [36]. Antibiosis occurs once ingestion has started and acts by slowing or inhibiting growth, lowering survival or reproductive capacity. For example, maize has resistance to lepidopteran pests, conferred by the defensive cysteine protease maize insect resistance 1 (Mir1-CP). Mir1-CP immediately localizes to the vasculature and adjacent tissues at herbivores’ feeding sites and interferes with the peritrophic matrix of lepidopteran larvae, resulting in reduced nutrient uptake and slowed larval growth [37,38]. Further, studies have confirmed that Purified Mir1-CP is directly toxic to several maize-feeding lepidopteran pests and also acts synergistically with sublethal concentrations of Cry2A-class *Bacillus thuringiensis* toxin. Mir1-CP permeabilizes the larval peritrophic matrix, facilitating Cry2A access to its binding sites on the midgut microvilli and consequently increasing toxin activity [37]. Therefore, the application of Mir1-CP to control caterpillar pests in maize by non-transgenic methods is feasible, and its use in other crops might be possible in combination with Bt toxins.

The effects of benzoxazinoids are also associated with post-ingestive resistance but vary across insect species and metabolites. The relative level of DIMBOA-glucoside (2,4-dihydroxy-7-methoxy-1,4-benzoxazin-3-one glucoside (DIMBOA-Glc)) versus 2-hydroxy-4,7-dimethoxy-1,4-benzoxazin-3-one glucoside (HDMBOA-Glc) has been linked to the resistance against chewing and sap-feeding insects and can result in trade-offs between caterpillar and aphid defence [9,39,40]. Among the other protein-based defences are digestive enzyme inhibitors. Kim et al. [41] have identified chymotrypsin inhibition as a post-ingestive resistance mechanism in several maize varieties functional against the Asian corn borer, *Ostrinia furnacalis*. Further, the maize serine protease inhibitor gene (SPI) is induced by both feeding with salicylic acid and *O. furnacalis*, and recombinant SPI proteins greatly inhibit larval development [24]. In line with these mechanisms, the leaf-based antibiosis effect of the tropical maize inbreds CML71 and CKSBL10008 is reflected in their ability to negatively impact FAW relative growth and increase larval development [27].

### 2.3. Tolerance

Tolerance is the capacity of a plant to maintain growth, physiological function, biomass, or yield at a given level of insect injury without necessarily reducing insect survival or reproduction [42]. Unlike antixenosis and antibiosis, tolerance primarily affects plant performance rather than the herbivore directly. It is mediated through compensatory processes such as maintenance or recovery of photosynthesis, redistribution of assimilates, antioxidant regulation, and regrowth following tissue loss [43]. Tolerance may therefore impose less direct selection for pest adaptation relative to resistance mechanisms that reduce herbivore fitness [44]. However, tolerant plants can sustain greater pest populations and their use should be integrated with management practices that limit pest abundance [42,43].

For example, the maize inbreds CML338 and Mp708 showed physiological tolerance to FAW injury. Following infestation, their photosynthetic rates and photosynthetic capacities remained comparable to those of uninfested plants. By contrast, photosynthesis declined in the susceptible lines AB24E and CML335 and in the resistant lines CML333 and CML336, indicating that resistance in CML333 and CML336 depends predominantly on mechanisms other than photosynthetic tolerance [45]. Compared with antixenosis and antibiosis, tolerance remains less frequently characterized in maize because its reliable identification requires simultaneous measurement of insect injury and plant performance across multiple infestation levels and environments [42]. Robust phenotyping must also distinguish true compensation from resistance-associated reductions in insect feeding or establishment.

### 2.4. Biochemical and Molecular Defences

Maize employs a wide range of biochemical compounds and signalling molecules to defend against insect pests across different feeding guilds, both directly by deterring herbivores and indirectly by influencing the behaviour and effectiveness of natural enemies, such as parasitoids and predators. For instance, the benzoxazinoids (BXs) in maize, a broad group of indole-derived defence metabolites that are key factors in plant–insect interactions, include, e.g., DIMBOA, which exhibits toxic effects against a range of insect pests, while its methoxylated derivative HDMBOA and their corresponding glucosides contribute to maize chemical defence, with HDMBOA reported to exhibit stronger toxicity against chewing lepidopteran larvae than DIMBOA [46,47,48]. In maize, BX1–BX5 constitute the core benzoxazinoid pathway, converting indole-3-glycerol phosphate through indole to 2,4-dihydroxy-1,4-benzoxazin-3-one (DIBOA). Following glucosylation of DIBOA by BX8 and BX9, BX6 and BX7 catalyse the final reactions leading to 2,4-dihydroxy-7-methoxy-1,4-benzoxazin-3-one glucoside (DIMBOA-Glc). Afterward, the O-methyltransferases BX10–BX12 convert DIMBOA-Glc into 2-hydroxy-4,7-dimethoxy-1,4-benzoxazin-3-one glucoside (HDMBOA-Glc) [49]. Conversion of DIMBOA to HDMBOA can enhance maize resistance to chewing caterpillars but reduce DIMBOA-dependent callose formation against aphids, indicating a trade-off between these defence responses [47].

Benzoxazinoids (BXs) are negatively regulated by the protein phosphatase ZmPP2C45, as the knockout significantly increases BXs accumulation and enhances resistance to insect herbivores through upregulation of BX biosynthetic genes. Mechanistically, ZmPP2C45 likely modulates the phosphorylation state of the transcription factor ZmBELL4, which suppresses BX biosynthesis, thereby identifying both as critical regulators and promising targets for improving maize insect resistance [50]. Beyond benzoxazinoids, maize also deploys volatile terpenoids as part of its inducible defence repertoire. These compounds serve a dual ecological function, either directly repelling herbivorous insects or indirectly enhancing plant protection by attracting their natural enemies. In maize, the enzyme terpene synthase 2 (TPS2) works together with the cytochrome P450 enzymes CYP92C5 and CYP92C6 to produce the defensive volatiles (E)-4,8-dimethyl-1,3,7-nonatriene (DMNT) and (E,E)-4,8,12-trimethyl-1,3,7,11-tridecatetraene (TMTT) [51]. Furthermore, phenolic compounds, including phenolic acids, flavonoids, tannins, stilbenes, and lignans, contribute to insect defence through several mechanisms.

Alkaloids are a separate class of nitrogen-containing defensive metabolites [52]. These include phenolic acids, stilbenes, lignans, and nitrogen-containing alkaloids. Flavonoids and tannins block insect digestive enzymes or bind to proteins to make them indigestible; lignin strengthens plant cell walls to make tissues tougher, and phenolic acids such as chlorogenic acid and caffeic acid can build up quickly within 24 h of insect attack to improve resistance [53]. The effectiveness of plant defences depends on the herbivore and its ability to adapt to host defences. In maize, benzoxazinoids can affect herbivores differently: DIMBOA suppresses Spodoptera littoralis, whereas *S. frugiperda* can detoxify it by converting DIMBOA to DIMBOA-Glc. In contrast, HDMBOA remains toxic to both species [47]. Interestingly, recent studies indicate that nutrient availability significantly modulates insect-defence pathways in plants. Nitrogen deficiency has been shown to enhance jasmonate-mediated defence responses and promote the accumulation of phenolic compounds. In contrast, excessive nitrogen often favours plant growth over defence, potentially increasing susceptibility to herbivores. Similarly, silicon nutrition can strengthen both physical and biochemical defence systems by stimulating JA signalling, protease inhibitor activity, and herbivore-induced volatile emissions that attractt natural enemies [54]. Herbivore attack triggers extensive genome-wide transcriptional reprogramming in plants, as revealed by RNA-seq studies that identify thousands of differentially expressed genes (DEGs) across species [55]. Studies have shown that feeding by Asian corn borer induces rapid changes in defence-related gene expression within ~2 h. In contrast, resistant lines rely more strongly on constitutive benzoxazinoid accumulation and show weaker inducible responses relative to susceptible lines [48]. Transcriptomic analyses of maize exposed to herbivory have identified differential expression of genes involved in cytochrome P450S, chitinases, protease inhibitors, peroxidases, terpene biosynthesis, and flavonoid metabolism, indicating coordinated activation of diverse chemical and defensive pathways [56,57].

**Figure 2:**
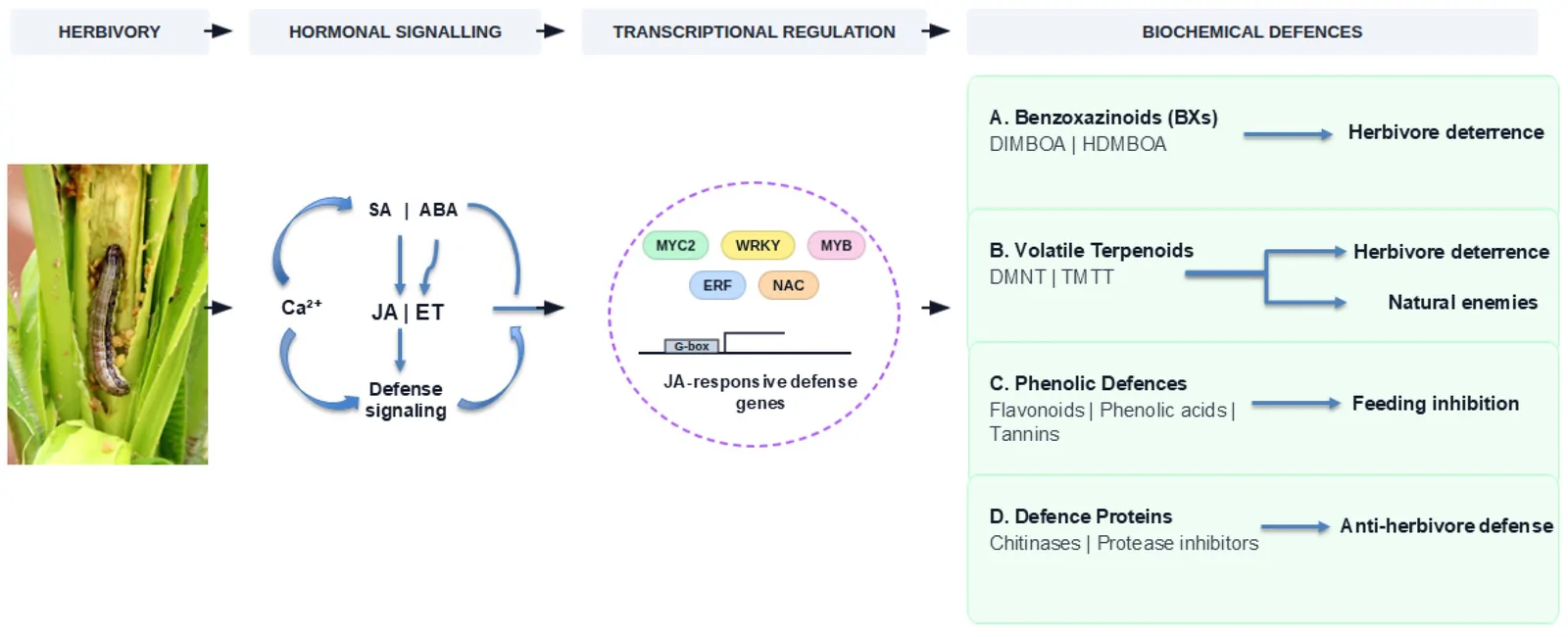
Biochemical and molecular defences in maize against insect herbivores. Abbreviations: Bxs, benzoxazinoids; DIMBOA, 2,4-dihydroxy-7-methoxy-1,4-benzoxazin-3-one; HDMBOA, 2-hydroxy-4,7-dimethoxy-1,4-benzoxazin-3-one; DMNT, (3E)-4,8-dimethyl-1,3,7-nonatriene; TMTT, (E,E)-4,8,12-trimethyl-1,3,7,11-tridecatetraene; SA, salicylic acid; ABA, abscisic acid; JA, jasmonic acid; ET, ethylene; Ca^2+^, calcium ion; MYC2, MYC2 transcription factor; WRKY, WRKY transcription factor family; MYB, MYB transcription factor family; ERF, ethylene response factor; NAC, NAC transcription factor family; G-box, G-box cis-regulatory element.

Regulation of these transcriptional changes involves conserved cis-elements, such as the G-box (CACGTG), which is recognized by jasmonic acid (JA)-responsive MYC2 transcription factors, as well as members of the WRKY, MYB, and ethylene-responsive factor families [58]. In maize, the MYC2 family members ZmMYC2a and ZmMYC2b have been functionally validated as partially redundant regulators of JA-mediated insect defence, benzoxazinoid biosynthesis, and volatile-terpene production [59]. Herbivory also induces transcriptional activators, including MYC and NAC proteins, as well as negative regulators of JA signalling, such as jasmonate ZIM-domain (JAZ) proteins and JA-associated MYC2-like (JAM) factors. Together with the induction of JA-catabolic genes, these regulatory components fine-tune the magnitude and duration of the defence response. Beyond transcription, post-translational modifications (PTMs) rapidly modulate the activity of defence proteins. Phosphorylation, targeted degradation, sequestration, redox regulation, and protein–protein interactions modulate transcription factor function, integrating signals from the JA, salicylic acid (SA), abscisic acid (ABA), and ethylene pathways that mediate responses to herbivore type and severity [29,60]. Small RNAs, such as microRNAs (miRNAs), also modulate hormone homeostasis by regulating genes involved in JA, SA, and ethylene biosynthesis and signalling, enabling rapid defence adjustments without large-scale transcriptional shifts [61]. Together, transcriptional reprogramming, PTMs, and miRNA-mediated regulation form a multilayered network that allows plants to mediate defences while minimizing trade-offs with growth and reproduction under insect infestation. The major biochemical and molecular defence mechanisms involved in maize resistance to insect herbivores are summarized in Figure 2.

## 3. Breeding Strategies for Insect-Resistant Maize

### 3.1. Conventional Breeding Strategies for Insect Resistance

Conventional breeding utilizes existing genetic variation to generate insect-resistant cultivars that can be adopted for IPM [2]. The primary step is screening a large number of germplasm, including elite lines, landraces, exotic germplasm, and wild relatives, for the presence of resistance alleles. The CIMMYT-developed germplasm, particularly the Multiple Insect Resistant Tropical (MIRT) and Multiple Borer Resistant (MBR) populations, as well as the Mp series and other resistant inbreds, continue to serve as excellent sources of resistance to FAW and stem borers [62].

Recurrent selection, pedigree breeding, backcrossing, hybrid breeding and population improvement are used by breeders to introduce resistance into elite backgrounds, depending on the genetic architecture of the target trait. Insect resistance is largely quantitative, so selection for accumulating favourable alleles, along with concomitant selection for grain yield, adaptation and other agronomic characters, remains challenging [3]. The latest results on FAW on maize revealed predominant non-additive gene action, including dominance and epistasis, suggesting recurrent selection as an effective strategy for improving insect resistance and yield stability in maize under variable pest pressure [3]. Therefore, the evaluation of a cultivar based on its phenotype is considered not only on the visual assessment of damage but also on the number of surviving insects and the yield produced [19].

Widening resistance is still vital to lasting control. Although there are several drawbacks, such as linkage drag, though wild relatives like teosinte, which are a good source of resistance; novel alleles have been shown in recent mapping studies [20,63]. Similarly, resistant germplasm has been developed by selecting germplasm with kernel hardness and pericarp characteristics that confer maize-weevil resistance, as well as biochemical defences that reduce maize-weevil infestation [12,13,64]. Furthermore, the native Mexican maize races Vandeño and Tabloncillo × Tuxpeño exhibit strong antixenosis, with significantly reduced feeding damage and oviposition. For instance, red spider mite oviposition averaged 25.67 ± 17.04 eggs on Celaya versus 7.00 ± 5.00 on the resistant Raton race [65]. Further, CML122, CML139, and Mp97:160 were identified as the maize lines that are most resistant to FAW, whereas FBLL and LH199 combined reduced corn earworm damage with favourable agronomic performance, highlighting their potential as valuable parental germplasm for insect-resistance breeding programs [66].

Although these advances have been made, traditional breeding methods are limited by polygenic inheritance of resistance, genotype-by-environment interactions, pest population variability, and the lack of uniform artificial infestation. As a result, today’s breeding practice increasingly combines conventional and molecular breeding, genomic prediction, and high-throughput phenotyping to promote genetic gain and the stability and durability of insect resistance across different environments [2,19,21]. Representative non-transgenic maize germplasms with resistance or tolerance to major insect pests are shown below (Table 1).

### 3.2. Quantitative Trait Locus (QTL) Mapping and Association Studies

Insect pest resistance in maize is polygenetic, as reported by QTLs mapping and association studies, and is highly dependent on the species, plant tissue, genetic background, and environment of the insect pest. A bi-parental mapping study identified eight QTLs for Mediterranean corn borer (*Sesamia nonagrioides*) in B73 × CML103, where favourable alleles of CML103 significantly decreased stalk tunnelling. In another study, QTLs were detected in A637 × A509 that simultaneously increased resistance and grain yield [73,76]. This type of colocalization of resistance loci with yield is of prime importance in breeding programs. Similarly, for fall armyworm, *Spodoptera frugiperda* and the southwestern corn borer, *Diatraea grandiosella*, also share resistance genes on chromosomes 6, 9, and 10 [77]. Major FAW-resistance QTLs have been identified in bins 4.06 and 9.03, whereas shoot-fly resistance QTLs associated with deadheart incidence and oviposition have been identified on chromosome 9 [74,78]. Further, QTLs have also been identified for resistance to maize weevil and corn leaf aphid, showing that different feeding guilds are controlled by different parts of the genome and partly shared [7,13].

GWAS studies have increased the number of alleles sampled from diverse maize germplasm and have identified many small-effect marker–trait associations, especially for FAW resistance [21]. A tissue-specific meta-analysis combined 86 meta-QTLs (24 in leaf, 42 in stem, and 20 in kernel resistance), some of which overlap with cell-wall-associated genes that reinforce cell walls [19]. A previous study identified QTLs associated with resistance to the Mediterranean stem borer and grain yield in selected recombinant inbred lines (RILs) of a maize MAGIC population that were tested in both testcrosses and per se. López-Malvar et al. [79] tested selected recombinant inbred lines (RILs) of a maize MAGIC population in both testcrosses and per se to evaluate the previously identified resistance QTLs for the Mediterranean stem borer and grain yield. Almost 75% of the QTLs tested for tunnel length, and about 10% of the QTLs tested for grain yield, were confirmed. QTLs in the genomic region 6.05–6.07 were consistently validated in multiple validation sets for resistance to stem tunnelling. MAS is better suited to validated, high-impact loci, while genomic selection is better suited to capture the dispersed, small-effect variation underlying most insect resistance traits. Table 2 summarizes representative QTL and marker–trait associations.

### 3.3. Marker-Assisted Selection (MAS)

MAS uses DNA markers linked to resistance genes or QTLs to accelerate the introgression and pyramiding of desirable alleles. During backcrossing, markers linked to target resistance loci can be used for foreground selection, while background and recombinant selection facilitate the recovery of the elite recurrent-parent genome and help minimize linkage drag. Consequently, MAS can reduce the number of generations required to recover desirable agronomic backgrounds while retaining target resistance alleles. Its effectiveness, however, is strongly dependent on the magnitude, stability, and transferability of the underlying genetic effects. So, MAS is most effective for resistance loci or QTLs with relatively large and consistent effects across genetic backgrounds and environments.

The application of MAS has produced promising results against several economically important pests. For example, MAS for resistance to the European corn borer (*Ostrinia nubilalis*) has been reported to complement conventional phenotypic selection effectively, while selection for resistance to the Mediterranean corn borer (*Sesamia nonagrioides*) increased resistance without significant adverse effects on grain yield [82,83]. In contrast, many QTLs associated with resistance to *Diatraea* spp. in tropical maize exhibit relatively small effects and strong genetic-background dependence, which limits the predictive value of MAS when it is applied in the absence of phenotypic validation [80]. The consideration of QTL effect size, genetic background, environmental stability, and QTL × environment interactions is the key to developing marker-based selection strategies.

Resistance-associated loci for FAW, stem borers, maize weevils, and other major insect pests have been identified through linkage mapping, genome-wide association studies (GWAS), and meta-QTL analyses. Several reported loci are associated with pathways involved in cell-wall architecture and other plant defence mechanisms, indicating potential targets for marker-assisted pyramiding [19,21]. Nevertheless, marker–trait associations identified in individual populations should not be considered immediately suitable for routine breeding applications. Their utility should be confirmed through independent populations, contrasting environments, pest populations and representative breeding or hybrid backgrounds before deployment.

Given the polygenic nature of native insect resistance in maize, MAS should therefore be integrated with, rather than replace, phenotypic selection and genomic selection. A complementary breeding framework can use validated large-effect loci for MAS, genomic selection to capture dispersed small-effect alleles, and standardized multi-environment phenotyping to assess resistance, yield, and other agronomic traits simultaneously. Such an integrated strategy is expected to improve the reliability of resistance breeding and facilitate the development of high-yielding maize cultivars with durable resistance to multiple insect pests [2,19].

### 3.4. Genomic Selection (GS) and Predictive Breeding

Genomic selection (GS) predicts the performance of resistance lines by estimating genomic estimated breeding values (GEBVs) from genome-wide markers. It is particularly promising for insect-resistance breeding because resistance and yield are complex, polygenic traits controlled by many small-effect loci that are difficult to capture through marker-assisted selection (MAS). It allows breeders to identify resistance lines without waiting for large-scale phenotyping screening. In contrast to genome-wide association studies (GWAS), which look for marker–trait associations, and MAS, which focuses on a small number of previously validated markers, GS utilises genome-wide variation as the trait under selection. This is especially relevant for FAW resistance, for which multi-environment phenotypic data can improve prediction by accounting for genotype × environment interactions [21]. Badji et al. [14] reported moderate-to-high prediction accuracy for FAW and maize weevil resistance and emphasized the greater importance of training-population relatedness and representativeness relative to prediction-model choice. Further, high heritability and genetic gain have been observed for several FAW-associated traits, implying the possibility of the application of genomic prediction for the development of resistance genotypes [71].

**Figure 3:**
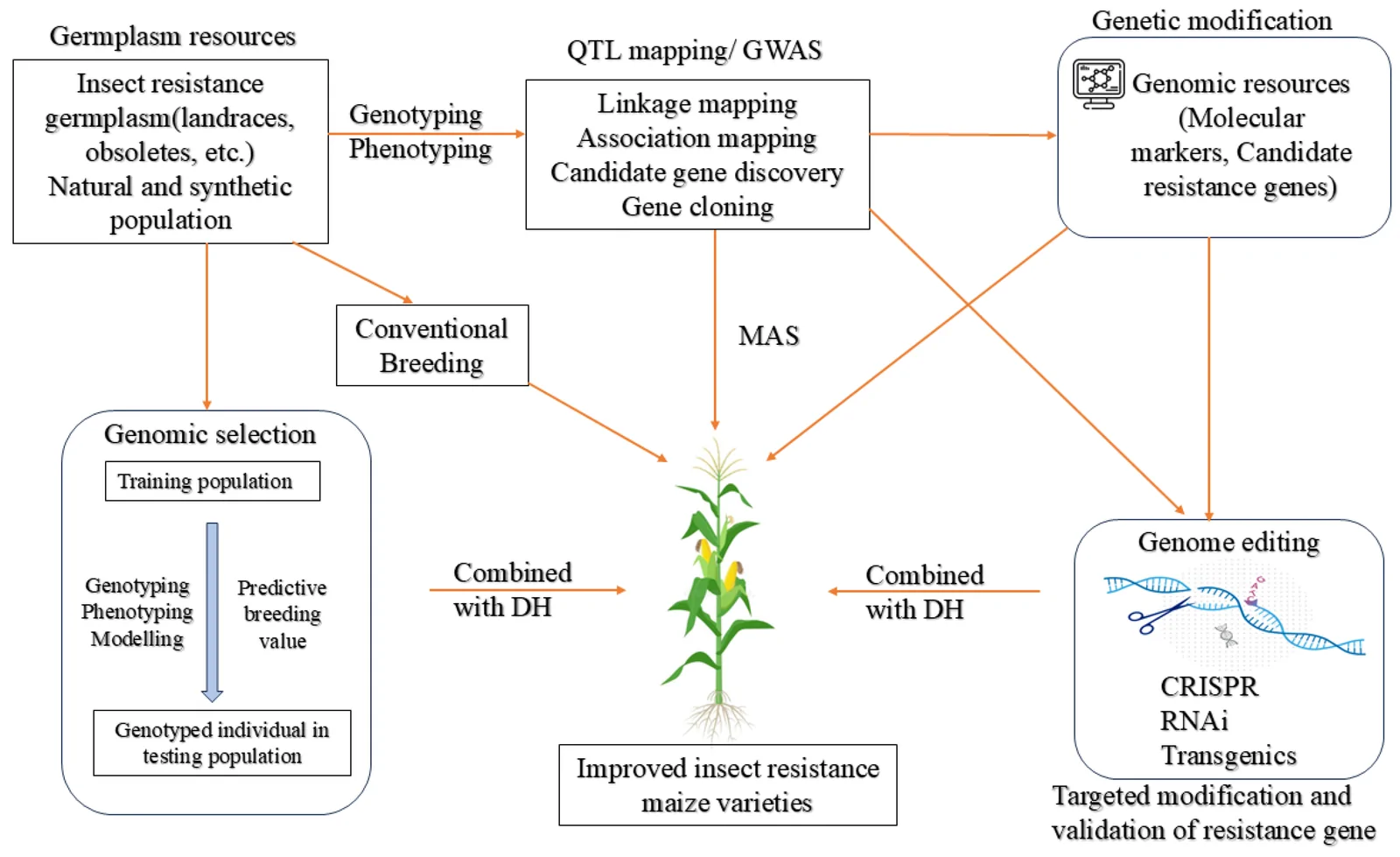
Breeding approaches for enhancing insect resistance in maize. Abbreviations: DH, doubled haploid; GWAS, genome-wide association study; MAS, marker-assisted selection; QTL, quantitative trait locus.

Studies have also shown that although several corn-borer QTLs were reproducible, their individual effects were generally too weak for routine MAS, where genome-wide prediction can be a more appropriate strategy for polygenic resistance [79]. Effective GS, therefore, requires standardised artificial infestation, replicated multi-environment phenotyping, continuously updated training populations, and models that incorporate G × E. Because resistance and yield may involve trade-offs, integrating GS with phenotypic selection and MAS can maximise genetic gain while maintaining durable resistance and agronomic performance. However, studies have also shown that for FAW infestation, incidence was not consistently associated with yield loss, as low larval densities and low damage severity did not significantly reduce maize productivity [84]. The major conventional and molecular breeding approaches for developing insect-resistant maize are summarized in Figure 3.

### 3.5. Transgenic Approach

Transgenic insect resistance extends the maize defence repertoire by introducing effectors that are absent from, or insufficiently expressed in, elite germplasm. The most established examples are insecticidal proteins from *Bacillus thuringiensis* (Bt). After ingestion, activated Cry or Vip proteins interact with susceptible insect midgut targets, disrupt epithelial integrity, and cause feeding cessation and mortality. Early Cry1Ab maize provided effective control of European corn borer, whereas later pyramids combined proteins such as Cry1A.105 and Cry2Ab2 to broaden pest coverage. However, stacking more proteins does not automatically confer durability. A useful pyramid requires each component to be effective against the same target pest, act through sufficiently independent pathways, show little cross-resistance, and ideally deliver high-dose exposure; otherwise, resistance to one component can erode the entire stack [85,86,87]. Representative transgenic strategies are summarized in Table 3.

RNA interference (RNAi) provides a mechanistically distinct, sequence-directed approach. In host-induced gene silencing (HIGS), maize produces double-stranded RNA (dsRNA) corresponding to an essential insect transcript. Following ingestion, the insect RNAi machinery processes dsRNA into small interfering RNAs that guide degradation of the target mRNA. Silencing *DvSnf7* in western corn rootworm established the feasibility of lethal plant-mediated RNAi [22]. This mechanism is deployed with Bt proteins in SmartStax PRO maize; field testing showed that the addition of *DvSnf7* dsRNA improved root protection, where resistance had reduced the performance of the Bt-only pyramid, although it did not remove the need for resistance management [91]. HIGS can also be multiplexed: polycistronic tRNA-artificial microRNA constructs targeting several genes increased silencing efficiency and impaired *Mythimna separata* development [92]. Thus, RNAi is most valuable as an independent mode of action rather than simply another component added to an already compromised pyramid.

RNAi performance remains strongly dependent on insect biology. Coleopterans are generally more responsive to orally delivered dsRNA than lepidopterans, in which alkaline gut conditions, extracellular nucleases, inefficient cellular uptake, endosomal sequestration, and limited systemic spread can weaken silencing [23]. Target selection must therefore consider transcript essentiality, expression in accessible tissues, sequence conservation within the target pest, and absence of consequential matches in non-target organisms. Construct design, dsRNA length and dose, plant processing of the trigger, and tissue- and developmental-stage-specific expression are equally important [23,93,94]. Constitutive HIGS may create prolonged selection pressure, whereas inducible or tissue-specific expression could concentrate exposure at the feeding site and reduce unnecessary environmental loading; these designs nevertheless require field validation across pest populations and crop backgrounds.

Spray-induced gene silencing (SIGS) is not itself a transgenic strategy but provides a useful comparator and potential complement. Topically applied dsRNA can be reformulated or replaced more readily than a plant-incorporated trait, yet field performance is constrained by ultraviolet degradation, rainfastness, uptake, persistence, and production cost. Nanoparticle, clay, lipid, and polymer carriers may improve delivery, but their environmental fates and non-target effects require case-specific assessment. Resistance-management plans must also distinguish continuous exposure from a constitutively expressed HIGS trait from the spatially and temporally intermittent exposure produced by RNA-based sprays [23,95]. Representative insect genome-editing targets associated with pest control are summarized in Table 4. Representative insect genome-editing targets associated with pest control are summarized in Table 4.

The central lesson from Bt and RNAi maize is that molecular novelty alone does not ensure durable resistance. Field-evolved resistance of FAW to Cry1F maize demonstrates how rapidly efficacy can decline under intense and spatially uniform selection [100]. Deployment should therefore couple mechanistically independent toxins or RNA targets with structured refuges, baseline susceptibility data, routine resistance monitoring, rapid remedial action, and locally adapted hybrids. Native quantitative resistance, crop rotation, biological control, and other integrated pest-management tactics can reduce the pest population exposed to any single transgene. Candidate events should also be assessed for expression stability, agronomic performance, food and feed safety, effects on natural enemies and non-target organisms, and efficacy across relevant pest genotypes and environments. Transgenic approaches are therefore best viewed as precisely engineered components of integrated resistance breeding, not as replacements for host-plant resistance or ecological pest management.

### 3.6. Genome Editing: A New Frontier in Maize Insect Resistance

Genome editing provides a complementary strategy for validating and modifying endogenous defence pathways in maize. In the CRISPR/Cas system, a single-guide RNA directs the nuclease to a defined genomic sequence, where it generates a double-strand break. Repair via non-homologous end joining commonly results in insertions or deletions that disrupt gene function, whereas the less frequent homology-directed repair pathway enables template-guided sequence replacement. Base editors and prime editors further expand the editing toolkit by allowing targeted nucleotide substitutions and small sequence modifications without conventional double-strand breaks. Optimised prime-editing systems have already generated heritable modifications in maize, although editing efficiency remains dependent on the target locus, construct design, and plant genotype [101,102,103,104].

Direct applications of genome editing to maize insect resistance remain limited but are beginning to provide compelling proofs of concept. Loss-of-function analysis of *ZmMYC2a* and *ZmMYC2b* showed that partially redundant jasmonate-responsive transcription factors regulate genes involved in benzoxazinoid and volatile-terpene biosynthesis. Double-mutant plants accumulated lower concentrations of defensive metabolites and were more susceptible to FAW and oriental armyworm, suggesting a role for ZmMYC2-mediated signalling in maize anti-herbivore defence [105]. More directly, targeted knockout of *ZmPP2C45*, which encodes a negative regulator of benzoxazinoid metabolism, increased total leaf benzoxazinoid content by more than threefold, enhanced expression of Bx biosynthetic genes, and restricted the growth of *S. frugiperda* and *Helicoverpa armigera*. These findings identify *ZmPP2C45* as a validated susceptibility gene and a promising target for resistance improvement, although its effects on yield, development, and resistance stability require multi-environment field evaluation [51].

Genome editing has also clarified the ecological functions of herbivore-induced volatiles. Disruption of maize genes required for the biosynthesis of (E)-4,8-dimethyl-1,3,7-nonatriene and (E,E)-4,8,12-trimethyl-1,3,7,11-tridecatetraene reduced the attraction of Asian corn borer to previously infested maize plants, confirming the assumption that these compounds are uniformly insect-repellent and demonstrating that volatile-mediated interactions depend on the receiving organism. Because the same volatiles may also influence predators and parasitoids, edited volatile profiles must be evaluated in multitrophic field systems rather than solely in herbivore assays [106].

The predominantly polygenic architecture of native maize insect resistance limits the value of relying exclusively on single-gene knockouts. Multiplex editing can instead be used to test and combine candidate genes identified through QTL mapping, genome-wide association studies, and multi-omics analyses. The BREEDIT platform demonstrated that multiple maize genes can be edited simultaneously and subsequently recombined through crossing, providing a scalable framework for dissecting complex quantitative traits [107]. Although this approach has not yet been applied directly to maize insect resistance, it could facilitate the functional evaluation of gene families and interacting defence pathways. Promoter editing, base editing, and prime editing may be particularly valuable because they can generate quantitative allelic variation rather than complete loss of gene function, potentially allowing defence expression to be optimised while minimising growth and yield penalties. CRISPR-based transcriptional activation and repression offer additional possibilities for tissue-specific or inducible regulation, but their use in maize insect-resistance breeding remains largely prospective [103,104,108].

Editing insect genomes represents a distinct application that should not be conflated with the development of gene-edited maize. Its current value lies primarily in functional validation of pest genes and elucidation of resistance mechanisms. For example, CRISPR/Cas9 knockout of *SfABCC2* in *S. frugiperda* conferred strong resistance to the Cry1F toxin, demonstrating that *SfABCC2* is a functional Bt receptor; disruption of this gene did not restore toxin susceptibility, as previously stated [109]. Similarly, deletion of three midgut double-stranded RNase genes reduced dsRNA degradation and increased RNA-interference efficiency in FAW, identifying an important determinant of lepidopteran RNAi responsiveness [110]. The establishment of an efficient, heritable CRISPR/Cas protocol in *O. nubilalis* has enabled experimental validation of genes associated with development, seasonal adaptation, reproduction, and Bt resistance [111]. These studies can support resistance monitoring and the discovery of new molecular targets, but they do not yet constitute field-ready genetic control strategies.

**Figure 4:**
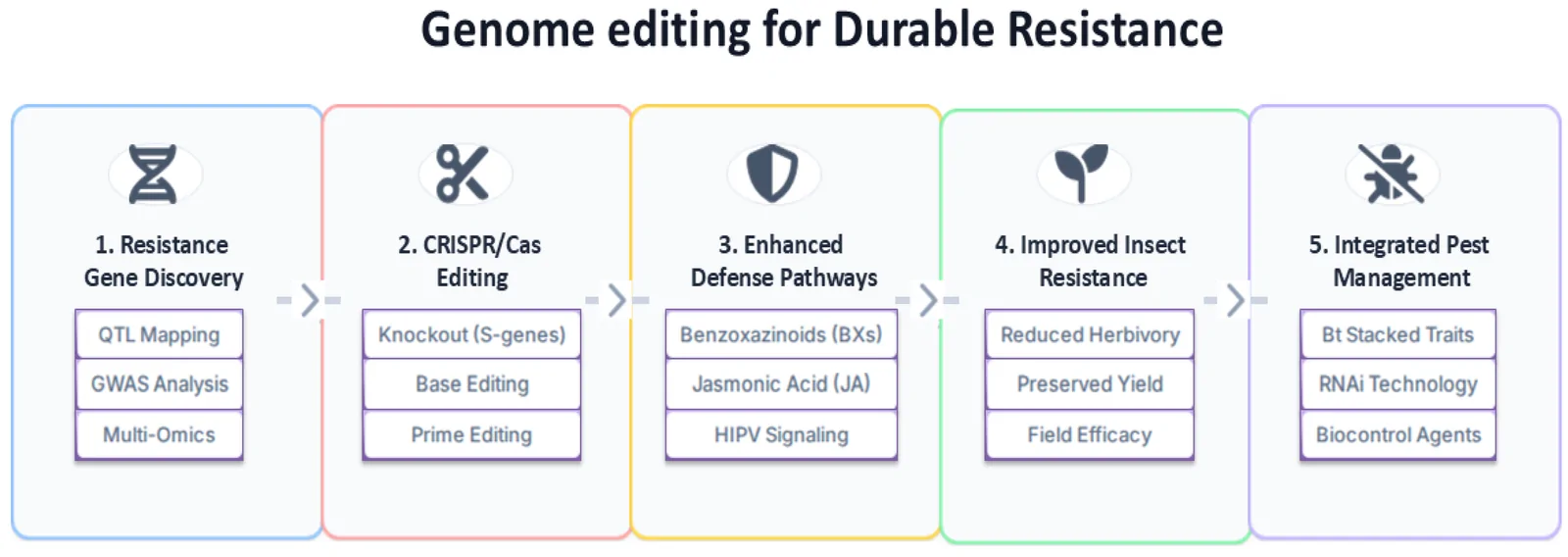
Integrated workflow for engineering insect resistance. The pipeline initiates with the discovery of genetic loci associated with natural resistance (1), followed by targeted manipulation using CRISPR/Cas technologies (2). These modifications aim to upregulate secondary metabolites and defence hormones (3), culminating in a robust phenotypic resistance against target pests (4). Finally, engineered traits are incorporated into broader, sustainable Integrated Pest Management (IPM) strategies (5).

Translation of genome editing into resistant maize cultivars will require several additional advances. Candidate genes must first be functionally validated and assessed for pleiotropic effects, particularly because defence signalling and specialized metabolism are closely connected to plant growth, reproduction, and responses to abiotic stress. Editing and regeneration systems must also become efficient across elite tropical and subtropical germplasm rather than remaining concentrated in a limited number of transformable genotypes. Edited lines should be evaluated across developmental stages, environments, pest populations, and feeding guilds, as well as for their effects on grain yield, natural enemies, and non-target organisms. For polygenic resistance, the most promising strategy is likely to combine precise editing of validated susceptibility or regulatory genes with conventional crossing, genomic selection, high-throughput phenotyping, and integrated pest management. Genome editing should therefore be viewed not as a replacement for breeding, but as a functional and precision-breeding tool for assembling complementary and evolutionarily durable resistance mechanisms (Figure 4) [51,104,108,112]. Efforts utilizing CRISPR/Cas9 technology to improve resistance against insect pests are presented in Table 5.

## 4. From Laboratory to Field: Achievement and Lessons from Bt Maize

The expression of insecticidal proteins isolated from the bacterium *Bacillus thuringiensis* (Bt) in maize is the most obvious example of a laboratory discovery with a wide-scale insect management application. Bt maize was commercially introduced in the mid-1990s and has been incorporated into locally adapted hybrids, each with one or more Cry and/or Vip proteins. Depending on the event, these proteins help maize resist lepidopteran pests such as European corn borer, Mediterranean corn borer, FAW, and corn earworm, as well as coleopteran pests such as western corn rootworm. Bt traits do not increase the intrinsic yield potential; instead, they minimize insect injury. Thus, their agronomic value relies on the presence of pests, the expression of toxins, the susceptibility of the target pests, the adaptation of the hybrid, and the crop management conditions [118,119].

Field experiments, farm surveys and meta-analyses show that Bt maize can lead to higher yield stability and farm profitability, and to reduced use of conventional insecticides. However, the sizes of these gains vary widely across production environments. A meta-analysis of 21 years of field testing showed higher grain yields with GM maize than with its near-isogenic counterparts and significantly reduced insect damage and mycotoxin levels [118]. In addition, a broader review of the literature, by Klümper and Qaim [120], reported that insect-resistant genetically modified crops achieved higher yields and reduced pesticide use compared with herbicide-tolerant crops. In Spain, yield and gross-margin benefits have been found when corn borers were a damaging pest, but benefits were only small when corn borers were not a damaging pest [121]. This resulted in higher yields and income, and lower insecticide expenses, for farmers using Bt maize compared with non-adopters [122]. Likewise, smallholder farmers in South Africa experienced yield benefits from Bt white maize when stem borer pressure was sufficiently high, but no significant yield benefits were observed when pressure was low [123]. The results have shown that in addition to event efficacy, these other factors, like pest ecology, seed cost, agronomic adaptation, and availability of proper hybrids to farmers, are also important for successful field translation.

Benefits from Bt maize may extend beyond the field. European corn borer (ECB), *Ostrinia nubilalis*, was suppressed nationwide, resulting in economic benefits for Bt- and non-Bt maize growers [119]. When *O. nubilalis* and *Helicoverpa* were suppressed by Bt field maize, subsequent landscape-scale analysis indicated that there was a corresponding reduction in insect damage and insecticide use in nearby vegetable crops, such as sweet corn, peppers and green beans [124]. Ear feeding can also be reduced by Bt, which may limit infection results from toxigenic fungi. Therefore, fumonisins, trichothecenes and other mycotoxins are often found at lower levels in the grain of insect-resistant maize than in comparable non-Bt hybrids, providing an additional benefit for food and feed quality [118].

The shift from laboratory efficacy to long-term performance in the field, however, is not permanent. A single insecticide protein may exert significant selection pressure on resistant pest genotypes when used continuously. In Brazil, the FAW developed field-evolved resistance to *Cry1F* maize, leading to significant larval survival on previously effective hybrids and constituting one of the fastest cases of efficacy loss under intensive selection pressure [100]. After several years of commercial cultivation of *Cry1Ab* maize in South Africa, resistance of the African maize stem borer, *Busseola fusca*, to Bt was observed [125]. Functional dominance undermines the high-dose/refuge (HDR) strategy because heterozygous offspring carrying resistance alleles can survive exposure to Bt crops when resistance is not completely recessive. This reduces the effectiveness of susceptible insects emerging from refuges in diluting resistance alleles, thereby accelerating the evolution and spread of resistance in pest populations [126,127]. Refuge-based resistance management, low initial resistance-allele frequencies, recessive inheritance of resistance, fitness costs, and incomplete resistance were identified as major factors contributing to the delayed evolution of insect resistance to Bt crops [128].

A recent study on over 85,000 observations from South Africa’s maize field trials also confirms that the initial yield benefit from first-generation Bt cultivars has been reduced due to the evolution of resistance; however, newer stacked cultivars have been associated with the recovery of yield benefit [129]. The findings here indicate that insecticidal effectiveness is dynamic; it can be lost through pest evolution after initial success.

As Bt maize is a key component of an integrated resistance management system, durable deployment requires a holistic approach. Baseline susceptibility data for specific pests and events, sufficient expression of the insecticidal protein, structured non-Bt refuges, monitoring of field efficacy, confirmation of suspected resistance, and quick remedial response should be part of good stewardship. Proteins should be stacked to provide independent and complementary action against the same target pest and to demonstrate low cross-resistance; stacking unrelated proteins (e.g., insect resistance and herbicide tolerance) does not necessarily delay insect adaptation. Furthermore, long-term durability of pyramids can be achieved only if resistance levels to one or more component proteins are high within the pest population. Recent on-farm testing of western corn rootworm has demonstrated that as western corn rootworm levels of resistance to pyramided Bt maize increase, the performance of pyramided Bt maize decreases, supporting the need to further deploy Bt maize in conjunction with other mechanisms of managing western corn rootworm in the field, such as crop rotation, biological control, native host-plant resistance and other population-suppressive techniques [130].

In summary, the efficacy of Bt maize comes not just from the introduction of an insecticidal gene, but from the synergistic combination of gene discovery, transformation, characterization of the modified event, the incorporation of the gene into adapted lines, regulatory assessment, seed availability, and resistance management. As in other new technologies, its history offers a model as well as a warning for new RNA-i and genome-editing technologies, as the high laboratory efficacy has led to significant agricultural benefits that will only be sustainable if evolutionary risk, farmer access, agronomic performance, and integrated pest management are taken into account from the outset of technology development.

## 5. Future Perspectives

To improve maize insect resistance in the future, a dynamic systems trait must be considered rather than a single gene, metabolite, or technology. The first step in the process is to translate the increasing catalogue of QTLs and marker–trait associations into validated causal variants and deployable resistance haplotypes. Pangenome resources, long-read sequencing and haplotype-based analyses should be used to capture structural variants and rare alleles not well-represented by single-reference SNP-based analyses and to provide diverse representation of tropical germplasm, landraces and teosinte. But the biggest challenge is to get the phenotyping right. Standardized artificial-infestation protocols, measurements at tissue- and developmental-stage-specific time frames and multi-environment assessments for insect performance, plant recovery, yield, and grain quality [19,21,63,131] are required for reliable gene discovery.

Functional genomics should relate candidate loci to defence mechanisms. Transcriptomic, proteomic, metabolomic and spatial-expression data can be integrated to identify regulatory networks involved in benzoxazinoid metabolism, protease inhibitors, cell-wall reinforcement and volatile signalling. Validation of these networks and the generation of desirable alleles are possible with CRISPR/Cas, but resistance to FAW and other pests has a predominantly polygenetic architecture, so single-gene knockouts are not very useful. Promoter editing, base and prime editing and multiplex approaches could be more effective, as they can control multiple defence components without reducing growth–defence trade-offs. The agronomic performance, resistance spectrum, environmental stability and impacts on beneficial and non-target organisms must be determined for edited materials before deployment [51,108].

But it is also essential to directly integrate climate resilience into resistance breeding. Understanding how nutrient availability influences defence signalling, specialised metabolite production, and multitrophic interactions may improve the prediction and deployment of durable insect resistance under diverse environmental conditions. The pressure from insects and the expression of defence mechanisms may vary with the crop’s developmental stage, drought, and temperature. Thus, selection should be done under combined biotic and abiotic stresses, not only under insect stress. However, genomic prediction, high-throughput phenotyping, and machine-learning techniques can help model these interactions, provided that datasets are harmonized and representative and are from multiple environments. Ultimately, the accuracy of prediction must be evaluated in terms of realized genetic gain and field performance, not statistical performance.

Complementary technologies under integrated pest management will be needed to provide durable resistance. Native quantitative resistance should be used creatively alongside Bt or RNAi traits, biological control, refuges, pest surveillance, and resistance monitoring. The deployment also needs to consider evolutionary changes in pest populations, farmer access to adapted seed, regional pest complexes, and more. Programs focused on single technologies are, therefore, less likely to produce lasting results as compared to breeding pipelines that integrate the discovery and validation of genes, gene function, predictive selection of resistant varieties, field testing, and ongoing resistance management from the beginning [2,87,95]. This integrated system might be the most feasible way forward in achieving high-yielding maize with stable resistance against multiple insect pests under climate change conditions.

## Figures and Tables

**Table 1:** Representative non-transgenic maize germplasms with resistance or tolerance to major insect pests.

Target Insect Pest	Resistant or Tolerant Germplasm	Breeding Relevance/Deployment Status	Reference(s)
Fall armyworm, *Spodoptera frugiperda*	CML71, CML 72, CML 338, CML 67, CKSBL10008, CML125, and Mp716	CML71 and CKSBL10008 showed favourable combining ability and are promising parents for the accumulation of native FAW resistance in tropical maize.	[3,27,67,68]
Fall armyworm, *Spodoptera frugiperda*	FAWTH2001, FAWTH2002, and FAWTH2003	Product-oriented hybrids for eastern and southern Africa. FAWTH2002 is being demonstrated in Kenya under the name Mjeshi and should be deployed as one component of IPM.	[69,70]
Fall armyworm, *Spodoptera frugiperda*	AS2001-20, AS2001-24, M1628-8, AS2106-63, and FAW2212	High-yielding under natural FAW pressure; promising germplasm for breeding and validation studies.	[71]
Fall armyworm, southwestern corn borer, and other lepidopteran pests	Mp708	A mechanistically characterized donor for functional studies and pyramiding endogenous protein defences with complementary resistance mechanisms.	[37,38]
Spotted stem borer, *Chilopartellus*, and African maize stem borer, *Busseolafusca*	CML559 (CKIRL08062) and CML560 (CKIRL08104)	CML559 belongs to the heterotic group AB, and CML560 to group B; their established combining ability facilitates their direct use as parents in hybrid breeding.	[72]
Mediterranean corn borer, *Sesamianonagrioides*	CML103	Useful for introducing tropical resistance alleles into temperate germplasm and for validating markers associated with stem-borer resistance.	[73]
Maize shoot fly, *Atherigonanaqvii*	CM143	A useful donor for spring-maize improvement; the major QTL require fine mapping and independent validation before routine MAS.	[74]
Corn leaf aphid, *Rhopalosiphummaidis*	Mo17 and Abe2	Strong donors for aphid-resistance fine mapping, diagnostic-marker development, and introgression into susceptible breeding backgrounds.	[7,75]
Maize weevil, *Sitophiluszeamais*	2000SYNEE-WSTR and TZBRELD3C5	Valuable donors for postharvest resistance breeding, subject to confirmation of grain quality, agronomic adaptation, and resistance stability under farm-storage conditions.	[12]
Asian corn borer, *Ostriniafurnacalis*	Ilmichal, P3394, and Kwangpyeongok	Useful sources for studying digestive-enzyme-mediated resistance and for identifying biochemical resistance factors.	[41]
Spotted stem borer, *C. partellus*	Resistant *Zea mays* ssp. *parviglumis* donor used in the LM13 × teosinte population	Demonstrates the value of teosinte for broadening resistance diversity; pre-breeding, interval refinement, and management of linkage drag are required before deployment.	[63]

**Table 2:** Representative quantitative trait locus (QTL) and association-mapping studies for insect resistance in maize.

Crosses	Mapping Population	Traits	No. of QTL	R^2^ (%)	Ref.
D06 × D408	F_3_ families	SDR; TL	5 SDR; 6 TL	≈50.0	[80]
B73 × CML103	RILs	TL; SDR; lodging; kernel and shank resistance	8	3.5–25.0	[73]
A637 × A509	RILs	TL; ear and stalk resistance; GY	12	NR	[76]
Eight-founder MAGIC	MAGIC RILs	TL; kernel resistance; GY	27 TL MTAs; 23 KR MTAs	3–9 per MTA	[81]
Mp704 × Mo17	F_2:3_ families	Leaf-feeding damage	7 FAW; 8 SWCB	14 FAW; 20 SWCB	[77]
Mp705 × Mp719	F_2:3_ families	Leaf-feeding damage	2 major	35.7, joint	[78]
Diverse CIMMYT panel	GWAS panel	Foliar and ear damage; ear rot; GY	56 MTAs	NR; minor effects	[21]
CM143 × CM144	F_2:3_ families	Oviposition; dead heart	19	15.03–18.89	[74]
Population 84 × Kilima	F_6_ RILs	GWL; AP; FP	15	14–51	[13]
B73 × Mo17	IBM RILs	Aphid reproduction	2	15; 27	[7]
B73 × Abe2	F_2:8_ RILs	Aphid-resistance score	1 major	40.12–55.17	[75]
LM13 × *Z. mays* ssp. *parviglumis*	F_6_ RILs	LIR; dead heart	4	9.00–21.35	[63]
Multiple populations	Meta-QTL analysis	Leaf, stem, and kernel resistance	86 MQTL	NA	[19]

Abbreviations: AP, adult progeny; FAW, fall armyworm (*Spodoptera frugiperda*); FP, flour production; GWL, grain-weight loss; GY, grain yield; KR, kernel resistance; LIR, leaf-injury rating; MTA, marker–trait association; MQTL, meta-QTL; RIL, recombinant inbred line; SDR, stalk-damage rating; SWCB, southwestern corn borer (*Diatraea grandiosella*); TL, tunnel length.

**Table 3:** Transgenic approaches for insect resistance in major crops.

Strategy	Gene(s) Introduced	Source Organism	Target Insect Pest(s)	Result	Reference
Insect resistance + Herbicide tolerance	*Cry1Ab*, *Cry2Ab*, *G10evo-epsps*	*Bacillus thuringiensis*	*Ostrinia furnacalis*, *Helicoverpa armigera*	Larval mortality	[88]
Bt Toxin Expression	*cry1Ab, cry1F, cry1A.105, cry2Ab2*	*Bacillus thuringiensis*	*Ostrinia nubilalis*, *Spodoptera frugiperda*, *Helicoverpa zea*	Reduced larval feeding injury; efficacy varied among proteins and target pests	[89]
Pyramided Bt traits	*cry1F, cry1A.105, cry2Ab2*	*Bacillus thuringiensis*	Multiple lepidopteran pests	Broader and prolonged protection; delayed resistance buildup	[85,87]
Protease Inhibitor Expression	*SerPIN*, *Cystatin*	Plant-derived	Generalist caterpillars	Impaired digestive enzyme activity in insects	[90]
RNA interference (RNAi)	dsRNA targeting *DvSnf7*, *v-ATPase*	Synthetic	*Diabrotica virgiferavirgifera* (Western corn rootworm)	Gene silencing and high larval mortality	[22]

**Table 4:** Insect genome editing for pest control.

Characteristics	Targeted Insect	Gene of Interest	References
Chitin synthase	Fall armyworm	*Chitin synthase* (*CHS2)*	[96]
Plant defence against herbivore	Fall armyworm	Glucose oxidase (GOX)	[97]
Detoxification enzyme control	Bollworm or pod borer	(Gene suppression) *CYP6AE* gene cluster	[98]
Body segmentation	Fall armyworm	(Mutation-induced alterations) *Sfabd-A*	[99]

**Table 5:** Utilizing CRISPR/Cas9 technology to improve resistance against insect pests.

Target Gene/Pathway	Maize Pest Targeted	Key Result	Reference
*SfruACY* (L-aminoacylase)	Fall armyworm (*Spodoptera frugiperda*)	Knockout larvae induced stronger jasmonate- and wound-responsive defences in maize and showed reduced growth on maize, with no growth effect on artificial diet.	[113]
Sex-specific *Sfdsx* isoforms	Fall armyworm (*S. frugiperda*)	Disruption caused genital abnormalities, reduced mating success, and sterility, identifying *Sfdsx* as a potential population-suppression target.	[114]
*SfPBAN*	Fall armyworm (*S. frugiperda*)	Editing reduced female attractiveness and fecundity, with mutant females producing no offspring when crossed with wild males.	[115]
*Sfabd-A*	Fall armyworm (*S. frugiperda*)	Knockout led to developmental abnormalities, reduced survival, and sterility, supporting its potential as a genetic control target.	[99]
*SfABCC2*	Fall armyworm (*S. frugiperda*)	Knockout conferred strong Cry1F resistance while leaving Vip3A susceptibility unchanged, confirming its role in Cry1F toxicity.	[109]
*SfCad*	Fall armyworm (*S. frugiperda*)	Knockout did not alter susceptibility to Cry1Ab or Cry1Fa, indicating that SfCad is not an essential receptor for these toxins.	[116]
*OfAbd-A* and *OfUbx*	Asian corn borer (*Ostrinia furnacalis*)	Disruption caused homeotic abnormalities and embryonic lethality, thereby identifying these developmental genes as potential genetic control targets.	[117]
*per*, *pdfr* and *ptth*	European corn borer (*Ostrinia nubilalis*)	Heritable editing was achieved; *per* affected adult-eclosion timing, whereas *ptth* mutants showed increased body weight and diapause.	[111]

## Data Availability

No new data were created or analyzed in this study. Data sharing is not applicable to this article.

## Abbreviations

The following abbreviations are used in this manuscript:

GBS	Genotyping-by-Sequencing
G-BLUP	Genomic Best Linear Unbiased Prediction
CRISPR	Clustered Regularly Interspaced Short Palindromic Repeats
sgRNA	Single Guide RNA
dCas9	Catalytically Dead Cas9
siRNA	Small Interfering RNA
SNPs	Single Nucleotide Polymorphisms
RNA-seq	RNA Sequencing
ISAAA	International Service for the Acquisition of Agri-Biotech Applications
DIMBOA	2,4-Dihydroxy-7-methoxy-1,4-benzoxazin-3-one
HDMBOA	2-Hydroxy-4,7-dimethoxy-1,4-benzoxazin-3-one

## References

[1] Erenstein O., Jaleta M., Sonder K., Mottaleb K., Prasanna B.M. (2022). Global maize production, consumption and trade: Trends and R&D implications. Food Secur..

[2] Prasanna B.M., Bruce A., Beyene Y., Makumbi D., Gowda M., Asim M., Martinelli S., Head G., Parimi S. (2022). Host plant resistance for fall armyworm management in maize: Relevance, status and prospects in Africa and Asia. Theor. Appl. Genet..

[3] Rohit, Ranjan R., Garg T., Jindal J. (2026). Deciphering the genetic architecture of fall armyworm resistance in maize. Cereal Res. Commun..

[4] Murenga M., Derera J., Mugo S., Tongoona P., Gichuru L. (2016). Evaluation of tropical maize inbred lines for resistance to two stem borer species, *Busseola fusca* and *Chilo partellus*. J. Plant Breed. Crop Sci..

[5] Munyiri S.W., Mugo S.N. (2017). Quantitative trait loci for resistance to spotted and African maize stem borers (*Chilo partellus* and *Busseola fusca*) in a tropical maize (*Zea mays* L.) population. Afr. J. Biotechnol..

[6] Pruter L.S., Weaver M., Brewer M.J. (2020). Overview of risk factors and strategies for management of insect-derived ear injury and aflatoxin accumulation for maize grown in subtropical areas of North America. J. Integr. Pest Manag..

[7] Betsiashvili M., Ahern K.R., Jander G. (2015). Additive effects of two quantitative trait loci that confer *Rhopalosiphum maidis* (corn leaf aphid) resistance in maize inbred line Mo17. J. Exp. Bot..

[8] Klein P., Smith C.M. (2020). Host plant selection and virus transmission by *Rhopalosiphum maidis* are conditioned by potyvirus infection in *Sorghum bicolor*. Arthropod-Plant Interact..

[9] Niu Y., Zhao X., Chao W.S., Lu P., Bai X., Mao T. (2023). Genetic variation, DIMBOA accumulation, and candidate gene identification in maize multiple insect-resistance. Int. J. Mol. Sci..

[10] López-Castillo L.M., Silva-Fernández S.E., Winkler R., Bergvinson D.J., Arnason J.T., García-Lara S. (2018). Postharvest insect resistance in maize. J. Stored Prod. Res..

[11] Koskei P., Bii C.C., Musotsi P., Muturi Karanja S. (2020). Postharvest storage practices of maize in Rift Valley and Lower Eastern regions of Kenya: A cross-sectional study. Int. J. Microbiol..

[12] Nwosu L.C., Adedire C.O., Ogunwolu E.O. (2015). Screening for new sources of resistance to *Sitophilus zeamais* Motschulsky (Coleoptera: Curculionidae) infestation in stored maize genotypes. J. Crop Prot..

[13] Castro-Álvarez F.F., William M., Bergvinson D.J., García-Lara S. (2015). Genetic mapping of QTL for maize weevil resistance in a RIL population of tropical maize. Theor. Appl. Genet..

[14] Badji A., Kwemoi D.B., Machida L., Okii D., Mwila N., Agbahoungba S., Kumi F., Ibanda A., Bararyenya A., Solemanegy M. (2020). Genetic basis of maize resistance to multiple insect pests: Integrated genome-wide comparative mapping and candidate gene prioritization. Genes.

[15] Tabashnik B.E., Fabrick J.A., Carrière Y. (2023). Global patterns of insect resistance to transgenic Bt crops: The first 25 years. J. Econ. Entomol..

[16] Painter R.H. (1951). Insect Resistance in Crop Plants.

[17] Tefera T., Mugo S., Beyene Y. (2016). Developing and deploying insect-resistant maize varieties to reduce pre- and post-harvest food losses in Africa. Food Secur..

[18] Borrego E.J., Kolomiets M.V. (2016). Synthesis and functions of jasmonates in maize. Plants.

[19] Badji A., Otim M., Machida L., Odong T., Kwemoi D.B., Okii D., Agbahoungba S., Mwila N., Kumi F., Ibanda A. (2018). Maize combined insect resistance genomic regions and their co-localization with cell wall constituents revealed by tissue-specific QTL meta-analyses. Front. Plant Sci..

[20] Maazou A.-R.S., Qiu J., Mu J., Liu Z. (2017). Utilization of wild relatives for maize (*Zea mays* L.) improvement. Afr. J. Plant Sci..

[21] Kamweru I., Anani B.Y., Beyene Y., Makumbi D., Adetimirin V.O., Prasanna B.M., Gowda M. (2022). Genomic analysis of resistance to fall armyworm (*Spodoptera frugiperda*) in CIMMYT maize lines. Genes.

[22] Baum J.A., Bogaert T., Clinton W., Heck G.R., Feldmann P., Ilagan O., Johnson S., Plaetinck G., Munyikwa T., Pleau M. (2007). Control of coleopteran insect pests through RNA interference. Nat. Biotechnol..

[23] Christiaens O., Whyard S., Vélez A.M., Smagghe G. (2020). Double-stranded RNA technology to control insect pests: Current status and challenges. Front. Plant Sci..

[24] Chen F., Chen L., Yan Z., Xu J., Feng L., He N., Guo M., Zhao J., Chen Z., Chen H. (2024). Recent advances of CRISPR-based genome editing for enhancing staple crops. Front. Plant Sci..

[25] Namata M.J., Xu J., Habyarimana E., Palakolanu S.R., Wang L., Li J. (2025). Genome editing in maize and sorghum: A comprehensive review of CRISPR/Cas9 and emerging technologies. Plant Genome.

[26] Smith C.M. (2005). Plant Resistance to Arthropods: Molecular and Conventional Approaches.

[27] Nuambote-Yobila O., Bruce A.Y., Okuku G.O., Marangu C., Makumbi D., Beyene Y., Mahungu N.-M., Prasanna B.M., Marion-Poll F., Calatayud P.-A. (2023). Assessment of resistance mechanisms to fall armyworm, *Spodoptera frugiperda*, in tropical maize inbred lines. Agronomy.

[28] Turlings T.C.J., Tumlinson J.H., Lewis W.J. (1990). Exploitation of herbivore-induced plant odors by host-seeking parasitic wasps. Science.

[29] Setotaw Y.B., Li J., Qi J., Ma C., Zhang M., Huang C., Wang L., Wu J. (2024). Salicylic acid positively regulates maize defenses against lepidopteran insects. Plant Divers..

[30] Costa E.N., Fernandes M.G., Medeiros P.H., Evangelista B.M.D. (2020). Resistance of maize landraces from Brazil to fall armyworm (Lepidoptera: Noctuidae) in the winter and summer seasons. Bragantia.

[31] Singh G., Xu J., Schaefer D., Day R., Wang Z., Zhang F. (2022). Maize diversity for fall armyworm resistance in a warming world. Crop Sci..

[32] Fathipour Y., Sedaratian A., El-Shemy H.A. (2013). Integrated management of *Helicoverpa armigera* in Soybean Cropping Systems. Soybean-Pest Resistance.

[33] McMullen M.D., Byrne P.F., Snook M.E., Wiseman B.R., Lee E.A., Widstrom N.W., Coe E.H. (1998). Quantitative trait loci and metabolic pathways. Proc. Natl. Acad. Sci. USA.

[34] Moawad S.S. (2014). Plant resistance to agricultural insect pests. Rev. Plant Stud..

[35] Oliveira W.B.d.S., Machado L.C., Posse S.C.P., Faita M.R., Santos H.J.G.d., Couto D.P.d., Bernardes C.d.O., Guilhen J.H.S., Ferreira A., Ferreira M.F.d.S. (2025). Natural resistance to *Spodoptera frugiperda* (J.E. Smith, 1797) (Lepidoptera: Noctuidae) in traditional maize varieties cultivated in southeast Brazil. Crop Sci..

[36] Khan Z., Mihm J.A. (1997). A review of entomological techniques and methods used to determine mechanisms and bases of stem borer resistance in maize. Insect Resistant Maize: Recent Advances and Utilization: Proceedings of an International Symposium Held at CIMMYT.

[37] Mohan S., Ma P.W.K., Williams W.P., Luthe D.S. (2008). A naturally occurring plant cysteine protease possesses remarkable toxicity against insect pests and synergizes *Bacillus thuringiensis* toxin. PLoS ONE.

[38] Lopez L., Camas A., Shivaji R., Ankala A., Williams P., Luthe D. (2007). Mir1-CP, a novel defense cysteine protease accumulates in maize vascular tissues in response to herbivory. Planta.

[39] Meihls L.N., Handrick V., Glauser G., Barbier H., Kaur H., Haribal M.M., Lipka A.E., Gershenzon J., Buckler E.S., Erb M. (2013). Natural variation in maize aphid resistance is associated with 2,4-dihydroxy-7-methoxy-1,4-benzoxazin-3-one glucoside methyltransferase activity. Plant Cell.

[40] Li B., Förster C., Robert C.A.M., Züst T., Hu L., Machado R.A.R., Berset J.-D., Handrick V., Knauer T., Hensel G. (2018). Convergent evolution of a metabolic switch between aphid and caterpillar resistance in cereals. Sci. Adv..

[41] Kim E.Y., Jung J.K., Kim I.H., Kim Y. (2022). Chymotrypsin is a molecular target of insect resistance of three corn varieties against the Asian corn borer, *Ostrinia furnacalis*. PLoS ONE.

[42] Peterson R.K., Varella A.C., Higley L.G. (2017). Tolerance: The forgotten child of plant resistance. PeerJ.

[43] Koch K.G., Chapman K., Louis J., Heng-Moss T., Sarath G. (2016). Plant tolerance: A unique approach to control hemipteran pests. Front. Plant Sci..

[44] Mitchell C., Brennan R.M., Graham J., Karley A.J. (2016). Plant defense against herbivorous pests: Exploiting resistance and tolerance traits for sustainable crop protection. Front. Plant Sci..

[45] Ni X., Da K., Buntin G.D., Brown S.L. (2008). Physiological basis of fall armyworm (Lepidoptera: Noctuidae) resistance in seedlings of maize inbred lines with varying levels of silk maysin. Fla. Entomol..

[46] Niemeyer H.M. (2009). Hydroxamic acids derived from 2-hydroxy-2H-1,4-benzoxazin-3(4H)-one: Key defense chemicals of cereals. J. Agric. Food Chem..

[47] Qi J., Malook S.U., Shen G., Gao L., Zhang C., Li J., Zhang J., Wang L., Wu J. (2018). Current understanding of maize and rice defense against insect herbivores. Plant Divers..

[48] Guo J., Qi J., He K., Wu J., Bai S., Zhang T., Zhao J., Wang Z. (2019). The Asian corn borer *Ostrinia furnacalis* feeding increases the direct and indirect defense of mid-whorl stage commercial maize in the field. Plant Biotechnol. J..

[49] Zhou H., Hua J., Li H., Song X., Luo S. (2023). Structurally diverse specialized metabolites of maize and their extensive biological functions. J. Cell. Physiol..

[50] Zhang S., Luo M., Deng R., Cai Y., Qi J., Ma C., Mei J., Li W., Liu W., Wang G. (2025). *ZmPP2C45* and *ZmBELL4* suppress maize biochemical defense against insect herbivores. New Phytol..

[51] Richter A., Schaff C., Zhang Z., Lipka A.E., Tian F., Köllner T.G., Schnee C., Preiß S., Irmisch S., Jander G. (2016). Characterization of biosynthetic pathways for the production of the volatile homoterpenes DMNT and TMTT in *Zea mays*. Plant Cell.

[52] Yu H., Zheng T., Li H., Chen J., Niu H., Tang X., Wang Z., Zhang X. (2025). Research progress on structural modification and action targets of plant-derived insecticidal active alkaloids. Pest Manag. Sci..

[53] Vasantha-Srinivasan P., Noh M.Y., Park K.B., Kim T.Y., Jung W.-J., Senthil-Nathan S., Han Y.S. (2025). Plant immunity to insect herbivores: Mechanisms, interactions, and innovations for sustainable pest management. Front. Plant Sci..

[54] Li Z.-X., Tan J.-F., Yao N., Xie R.-H. (2024). From trade-off to synergy: How nutrient status modulates plant resistance to herbivorous insects?. Adv. Biotechnol..

[55] Wang H., Li S., Teng S., Liang H., Xin H., Gao H., Huang D., Lang Z. (2017). Transcriptome profiling revealed novel transcriptional regulators in maize responses to *Ostrinia furnacalis* and jasmonic acid. PLoS ONE.

[56] Qi J., Sun G., Wang L., Zhao C., Hettenhausen C., Schuman M.C., Baldwin I.T., Li J., Song J., Liu Z. (2016). Oral secretions from *Mythimna separata* insects specifically induce defense responses in maize as revealed by high-dimensional biological data. Plant Cell Environ..

[57] Lawrence S.D., Novak N.G., Kayal W.E., Ju C.J.-T., Cooke J.E.K. (2012). Root herbivory: Molecular analysis of the maize transcriptome upon infestation by southern corn rootworm, *Diabrotica undecimpunctata howardi*. Physiol. Plant..

[58] Li Z., Huang Y., Shen Z., Wu M., Huang M., Hong S.-B., Xu L., Zang Y. (2024). Advances in functional studies of plant MYC transcription factors. Theor. Appl. Genet..

[59] Li S., Ma C., Li S., Zhang M., Zhang C., Qi J., Wang L., Wu X., Li J., Wu J. (2025). Mitogen-activated protein kinase 4 phosphorylates MYC2 transcription factors to regulate jasmonic acid signaling and herbivory responses in maize. Plant Physiol..

[60] Caarls L., Pieterse C.M.J., Van Wees S.C.M. (2015). How salicylic acid takes transcriptional control over jasmonic acid signaling. Front. Plant Sci..

[61] Luo C., Bashir N.H., Li Z., Liu C., Shi Y., Chu H. (2024). Plant microRNAs regulate the defense response against pathogens. Front. Microbiol..

[62] Matova P.M., Kamutando C.N., Magorokosho C., Kutywayo D., Gutsa F., Labuschagne M. (2020). Fall armyworm invasion, control practices and resistance breeding in sub-Saharan Africa. Crop Sci..

[63] Kaur R., Kaur G., Navpreet, Ashmita, Jindal J., Kumar R., Vikal Y., Sharma P. (2025). Gaining insights into genomic regions associated with *Chilo partellus* resistance in teosinte-derived maize population. Front. Genet..

[64] García-Lara S., Bergvinson D.J. (2014). Phytochemical and nutraceutical changes during recurrent selection for storage pest resistance in tropical maize. Crop Sci..

[65] Rocandio-Rodríguez M., Torres-Castillo J.A., Juárez-Aragón M.C., Chacón-Hernández J.C., Moreno-Ramírez Y.d.R., Mora-Ravelo S.G., Delgado-Martínez R., Hernández-Juárez A., Heinz-Castro R.T.Q., Reyes-Zepeda F. (2022). Evaluation of resistance of eleven maize races (*Zea mays* L.) to the red spider mite (*Tetranychus merganser* Boudreaux). Plants.

[66] Woolfolk S.W., Jeffers D.P., Read Q.D., Hawkins L.K., Babiker E.M. (2026). Assessing varied maize germplasm lines for resistance to fall armyworm and corn earworm (Lepidoptera: Noctuidae) with agronomic quality consideration. Fla. Entomol..

[67] Nuambote-Yobila O., Bruce A.Y., Makumbi D., Ogola G.O., Marangu C., Beyene Y., Mahungu N.-M., Prasanna B.M., Marion-Poll F., Calatayud P.-A. (2025). Combining ability for resistance to fall armyworm, *Spodoptera frugiperda*, in tropical maize inbred lines. Phytoparasitica.

[68] Kaur K., Cheema H.K., Jindal J., Garg T., Singh G., Atri A. (2024). Categorization of resistance against the fall armyworm, *Spodoptera frugiperda* (J.E. Smith), in select maize inbred lines. Crop Prot..

[69] Amegbor I.K., Adu G.B., Nelimor C., Prasanna B.M., Beyene Y., Chivasa W., Gethi J.G., Togola A., Jamal-deen A., Adogoba D.S. (2025). Assessment of fall armyworm-tolerant maize hybrids for sustainable maize production in sub-Saharan Africa. Phytoparasitica.

[70] International Maize and Wheat Improvement Center (CIMMYT) Announcing CIMMYT-Derived Fall Armyworm-Tolerant Elite Maize Hybrids for Eastern and Southern Africa. https://www.cimmyt.org/news/announcing-cimmyt-derived-fall-armyworm-tolerant-elite-maize-hybrids-for-eastern-and-southern-africa/.

[71] Obok E., Ulafor J., Iwo G., An P., Liu J., Eneji A. (2025). Field response and genetic variability analyses of maize (*Zea mays* L.) hybrids and commercial varieties under natural fall armyworm (*Spodoptera frugiperda*) infestation in lowland tropical ecology. Maydica.

[72] International Maize and Wheat Improvement Center (CIMMYT) CIMMYT Releases 22 New Maize Inbred Lines for the Tropics and Subtropics. https://www.cimmyt.org/news/cimmyt-releases-22-new-maize-inbred-lines-for-the-tropics-and-subtropics/.

[73] Samayoa L.F., Malvar R.A., McMullen M.D., Butrón A. (2015). Identification of QTL for resistance to Mediterranean corn borer in a maize tropical line to improve temperate germplasm. BMC Plant Biol..

[74] Vikal Y., Kaur A., Jindal J., Kaur K., Pathak D., Garg T., Singh A., Singh P., Yadav I. (2020). Identification of genomic regions associated with shoot fly resistance in maize and their syntenic relationships in the sorghum genome. PLoS ONE.

[75] Wang T., Wang K., Wang C., Zhao Y., Tao Z., Li J., Wang L., Shi J., Huang S., Xie C. (2023). Combining quantitative trait locus mapping with multiomics profiling reveals genetic control of corn leaf aphid (*Rhopalosiphum maidis*) resistance in maize. J. Exp. Bot..

[76] Jiménez-Galindo J.C., Ordás B., Butrón A., Samayoa L.F., Malvar R.A. (2017). QTL mapping for yield and resistance against Mediterranean corn borer in maize. Front. Plant Sci..

[77] Brooks T.D., Willcox M.C., Williams W.P., Buckley P.M. (2005). Quantitative trait loci conferring resistance to fall armyworm and southwestern corn borer leaf feeding damage. Crop Sci..

[78] Womack E.D., Williams W.P., Smith J.S., Warburton M.L., Bhattramakki D. (2020). Mapping quantitative trait loci for resistance to fall armyworm (Lepidoptera: Noctuidae) leaf-feeding damage in maize inbred Mp705. J. Econ. Entomol..

[79] López-Malvar A., Reséndiz-Ramírez Z., Butrón A., Jiménez-Galindo J.C., Revilla P., Malvar R.A. (2024). Validation of QTLs associated with corn borer resistance and grain yield: Implications in maize breeding. Front. Plant Sci..

[80] Bohn M., Groh S., Khairallah M.M., Hoisington D.A., Utz H.F., Melchinger A.E. (2001). Re-evaluation of the prospects of marker-assisted selection for improving insect resistance against *Diatraea spp.* in tropical maize by cross-validation and independent validation. Theor. Appl. Genet..

[81] Jiménez-Galindo J.C., Malvar R.A., Butrón A., Santiago R., Samayoa L.F., Caicedo M., Ordás B. (2019). Mapping of resistance to corn borers in a MAGIC population of maize. BMC Plant Biol..

[82] Flint-Garcia S.A., Darrah L.L., McMullen M.D., Hibbard B.E. (2003). Phenotypic versus marker-assisted selection for stalk strength and second-generation European corn borer resistance in maize. Theor. Appl. Genet..

[83] Samayoa L.F., Butrón A., Malvar R.A. (2019). Usefulness of marker-assisted selection to improve maize for increased resistance to *Sesamia nonagrioides* attack with no detrimental effect on yield. Ann. Appl. Biol..

[84] Mpawenimana A., Kahuthia-Gathu R., Mwangi M., Agbodzavu M.K., Abang A.F., Lamboni Y., Hitimana C., Kantungeko D., Kiiti P.M., Fiaboe K.K.M. (2026). Efficacy of selected environmentally friendly management options against the fall armyworm *Spodoptera frugiperda* (Lepidoptera: Noctuidae) and their cost-effectiveness on maize *Zea mays* in Burundi. Int. J. Trop. Insect Sci..

[85] Carrière Y., Crickmore N., Tabashnik B.E. (2015). Optimizing pyramided transgenic Bt crops for sustainable pest management. Nat. Biotechnol..

[86] Rule D.M., Nolting S.P., Prasifka P.L., Storer N.P., Hopkins B.W., Scherder E.F., Siebert M.W., Hendrix W.H. (2014). Efficacy of pyramided Bt proteins Cry1F, Cry1A.105, and Cry2Ab2 expressed in SmartStax corn hybrids against lepidopteran insect pests in the northern United States. J. Econ. Entomol..

[87] Tabashnik B.E., Carrière Y., Wu Y., Fabrick J.A. (2023). Global perspectives on field-evolved resistance to transgenic Bt crops: A special collection. J. Econ. Entomol..

[88] Zhu C., Qi L., Yu Y., Zhang X., Ying J., Ye Y., Shen Z. (2025). Molecular characterization and assessment of insect resistance of transgenic maize ZDRF-8. Plants.

[89] Fitches E., Edwards M.G., Mee C., Grishin E., Gatehouse A.M.R., Edwards J.P., Gatehouse J.A. (2004). Fusion proteins containing insect-specific toxins as pest control agents: Snowdrop lectin delivers fused insecticidal spider venom toxin to insect haemolymph following oral ingestion. J. Insect Physiol..

[90] Gatehouse J.A. (2011). Prospects for using proteinase inhibitors to protect transgenic plants against attack by herbivorous insects. Curr. Protein Pept. Sci..

[91] Reinders J.D., Moar W.J., Head G.P., Hassan S., Meinke L.J. (2023). Effects of SmartStax and SmartStax PRO maize on western corn rootworm (*Diabrotica virgifera virgifera* LeConte) larval feeding injury and adult life history parameters. PLoS ONE.

[92] Bao W., Li A., Zhang Y., Diao P., Zhao Q., Yan T., Zhou Z., Duan H., Li X., Wuriyanghan H. (2021). Improvement of host-induced gene silencing efficiency via polycistronic-tRNA-amiR expression for multiple target genes and characterization of RNAi mechanism in *Mythimna separata*. Plant Biotechnol. J..

[93] Koch A., Wassenegger M. (2021). Host-induced gene silencing: Mechanisms and applications. New Phytol..

[94] ZandKarimi H., Innes R.W. (2022). Molecular mechanisms underlying host-induced gene silencing. Plant Cell.

[95] Narva K.E., Toprak U., Alyokhin A., Groves R.L., Jurat-Fuentes J.L., Moar W.J., Nauen R., Whipple S.D., Head G. (2025). Insecticide resistance management scenarios differ for RNA-based sprays and traits. Insect Mol. Biol..

[96] Liu Z., Liao C., Zou L., Jin M., Shan Y., Quan Y., Yao H., Zhang L., Wang P., Liu Z. (2024). Retrotransposon-mediated disruption of a chitin synthase gene confers insect resistance to *Bacillus thuringiensis* Vip3Aa toxin. PLoS Biol..

[97] Gao B., Li B., Yuan J., Shi Z., Zheng X., Wang G. (2024). *Spodoptera frugiperda* salivary glucose oxidase reduces the release of green leaf volatiles and increases terpene emission from maize. Insects.

[98] Wang J., Yang M., Song Y., Acevedo F.E., Hoover K., Zeng R., Felton G.W. (2018). Gut-associated bacteria of Helicoverpa zea indirectly trigger plant defenses in maize. J. Chem. Ecol..

[99] Wu K., Shirk P.D., Taylor C.E., Furlong R.B., Shirk B.D., Pinheiro D.H., Siegfried B.D. (2018). CRISPR/Cas9-mediated knockout of the abdominal-A homeotic gene in fall armyworm moth, *Spodoptera frugiperda*. PLoS ONE.

[100] Farias J.R., Andow D.A., Horikoshi R.J., Sorgatto R.J., Fresia P., dos Santos A.C., Omoto C. (2014). Field-evolved resistance to Cry1F maize by *Spodoptera frugiperda* (Lepidoptera: Noctuidae) in Brazil. Crop Prot..

[101] Barrangou R., Doudna J.A. (2016). Applications of CRISPR technologies in research and beyond. Nat. Biotechnol..

[102] Swain S., Debnath S., Khamassi K., Samantara K., Ram P., Kumar A., Mahawer A.K., Kaul T. (2025). Harnessing genome editing for the advancement of underutilized crops: A critical review highlighting current progress, challenges and future prospects. Plant Gene.

[103] Qiao D., Wang J., Lu M.-H., Xin C., Chai Y., Jiang Y., Sun W., Cao Z., Guo S., Wang X.-C. (2023). Optimized prime editing efficiently generates heritable mutations in maize. J. Integr. Plant Biol..

[104] Tuncel A., Pan C., Clem J.S., Liu D., Qi Y. (2025). CRISPR-Cas applications in agriculture and plant research. Nat. Rev. Mol. Cell Biol..

[105] Ma C., Li R., Sun Y., Zhang M., Li S., Xu Y., Song J., Li J., Qi J., Wang L. (2023). ZmMYC2s play important roles in maize responses to simulated herbivory and jasmonate. J. Integr. Plant Biol..

[106] Zhao M., Huang S., Zhang Q., Wei Y., Tao Z., Wang C., Zhao Y., Zhang X., Dong J., Wang L. (2024). The plant terpenes DMNT and TMTT function as signaling compounds that attract Asian corn borer (*Ostrinia furnacalis*) to maize plants. J. Integr. Plant Biol..

[107] Lorenzo C.D., Debray K., Herwegh D., Develtere W., Impens L., Schaumont D., Vandeputte W., Aesaert S., Coussens G., De Boe Y. (2023). BREEDIT: A multiplex genome editing strategy to improve complex quantitative traits in maize. Plant Cell.

[108] Hernandes-Lopes J., Pinto M.S., Vieira L.R., Monteiro P.B., Gerasimova S.V., Nonato J.V.A., Bruno M.H.F., Vikhorev A., Rausch-Fernandes F., Gerhardt I.R. (2023). Enabling genome editing in tropical maize lines through an improved, morphogenic regulator-assisted transformation protocol. Front. Genome Ed..

[109] Jin M.-H., Tao J.-H., Li Q., Cheng Y., Sun X.-X., Wu K.-M., Xiao Y.-T. (2021). Genome editing of the *SfABCC2* gene confers resistance to Cry1F toxin from *Bacillus thuringiensis* in *Spodoptera frugiperda*. J. Integr. Agric..

[110] Koo J., Zhu G.-H., Palli S.R. (2024). CRISPR-Cas9-mediated dsRNase knockout improves RNAi efficiency in the fall armyworm. Pestic. Biochem. Physiol..

[111] Dayton J.N., Tran T.T., Saint-Denis E., Dopman E.B. (2025). Efficient CRISPR/Cas9-mediated genome editing in the European corn borer, *Ostrinia nubilalis*. Insect Mol. Biol..

[112] Tyagi S., Kumar R., Kumar V., Won S.Y., Shukla P. (2021). Engineering disease-resistant plants through CRISPR-Cas9 technology. GM Crops Food.

[113] Li B., Luo M., Liu X., Shi W., Qi J., Zhou S., Wang G. (2025). The *Spodoptera frugiperda* L-aminoacylase degrades fatty acid-amino acid conjugates and promotes larval growth on *Zea mays*. Commun. Biol..

[114] Gu J., Wang J., Bi H., Li X., Merchant A., Zhang P., Zhang Q., Zhou X. (2022). CRISPR/Cas9-mediated mutagenesis of sex-specific doublesex splicing variants leads to sterility in *Spodoptera frugiperda*, a global invasive pest. Cells.

[115] Ashok K., Bhargava C.N., Asokan R., Pradeep C., Pradhan S.K., Kennedy J.S., Balasubramani V., Murugan M., Jayakanthan M., Geethalakshmi V. (2023). CRISPR/Cas9-mediated editing of pheromone biosynthesis activating neuropeptide (PBAN) gene disrupts mating in the fall armyworm, *Spodoptera frugiperda* (J.E. Smith) (Lepidoptera: Noctuidae). 3 Biotech.

[116] Zhang J., Jin M., Yang Y., Liu L., Yang Y., Gómez I., Bravo A., Soberón M., Xiao Y., Liu K. (2020). The cadherin protein is not involved in susceptibility to *Bacillus thuringiensis* Cry1Ab or Cry1Fa toxins in *Spodoptera frugiperda*. Toxins.

[117] Bi H., Merchant A., Gu J., Li X., Zhou X., Zhang Q. (2022). CRISPR/Cas9-mediated mutagenesis of *Abdominal-A* and *Ultrabithorax* in the Asian corn borer, *Ostrinia furnacalis*. Insects.

[118] Pellegrino E., Bedini S., Nuti M., Ercoli L. (2018). Impact of genetically engineered maize on agronomic, environmental and toxicological traits: A meta-analysis of 21 years of field data. Sci. Rep..

[119] Hutchison W.D., Burkness E.C., Mitchell P.D., Moon R.D., Leslie T.W., Fleischer S.J., Abrahamson M., Hamilton K.L., Steffey K.L., Gray M.E. (2010). Areawide suppression of European corn borer with Bt maize reaps savings to non-Bt maize growers. Science.

[120] Klümper W., Qaim M. (2014). A meta-analysis of the impacts of genetically modified crops. PLoS ONE.

[121] Gómez-Barbero M., Berbel J., Rodríguez-Cerezo E. (2008). Bt corn in Spain: The performance of the EU’s first GM crop. Nat. Biotechnol..

[122] Yorobe J.M., Quicoy C.B. (2006). Economic impact of Bt corn in the Philippines. Philipp. Agric. Sci..

[123] Gouse M., Pray C.E., Schimmelpfennig D., Kirsten J. (2006). Three seasons of subsistence insect-resistant maize in South Africa: Have smallholders benefited?. AgBioForum.

[124] Dively G.P., Venugopal P.D., Bean D., Whalen J., Holmstrom K., Kuhar T.P., Doughty H.B., Patton T., Cissel W., Hutchison W.D. (2018). Regional pest suppression associated with widespread Bt maize adoption benefits vegetable growers. Proc. Natl. Acad. Sci. USA.

[125] Van Rensburg J.B.J. (2007). First report of field resistance by the stem borer, *Busseola fusca* (Fuller), to Bt-transgenic maize. S. Afr. J. Plant Soil.

[126] Campagne P., Kruger M., Pasquet R., Le Ru B., Van den Berg J. (2013). Dominant inheritance of field-evolved resistance to Bt corn in *Busseola fusca*. PLoS ONE.

[127] Campagne P., Smouse P.E., Pasquet R., Silvain J.F., Le Ru B., Van den Berg J. (2016). Impact of violated high-dose refuge assumptions on evolution of Bt resistance. Evol. Appl..

[128] Tabashnik B.E., Carrière Y., Dennehy T.J., Morin S., Sisterson M.S., Roush R.T., Shelton A.M., Zhao J.-Z. (2003). Insect resistance to transgenic Bt crops: Lessons from the laboratory and field. J. Econ. Entomol..

[129] Tack J., Cooper C.F., Nalley L.L., Chaminuka P., Maali S., Farmer E.E., Gore M.A. (2026). Quantifying yield losses from Bt resilience among maize cultivars in South Africa. Nat. Commun..

[130] Meinke L.J., Reinders J.D., Dang T.B., Krumm J.T., Pilcher C.D., Carroll M.W., Head G.P. (2024). Resistance management and integrated pest management insights from deployment of a Cry3Bb1 + Gpp34Ab1/Tpp35Ab1 pyramid in a resistant western corn rootworm landscape. PLoS ONE.

[131] Warburton M.L., Woolfolk S.W., Smith J.S., Hawkins L.K., Castano-Duque L.M., Lebar M.D., Williams W.P. (2023). Genes and genetic mechanisms contributing to fall armyworm resistance in maize. Plant Genome.

